# Supplementary material for: Viral mimicry redirects immunosuppressed colorectal tumour landscapes towards a proinflammatory and CMS1-like regenerative state
Source: Commun Biol. 2026 May 26;9:1119. doi: 10.1038/s42003-026-10295-9 (PMC13486658; doi:10.1038/s42003-026-10295-9)
Supplement: Supplementary file 1 [file 42003_2026_10295_MOESM1_ESM.pdf]

nature portfolio

Peer Review File

Viral mimicry redirects immunosuppressed colorectal tumour landscapes towards a proinflammatory and CMS1-like regenerative state

Corresponding Author: Dr Philip Dunne

This file contains all reviewer reports in order by version, followed by all author rebuttals in order by version.

Version 0:

Reviewer comments:

Reviewer #1

(Remarks to the Author)
This manuscript, submitted to Communications Biology, investigates the effects of the viral mimetic poly(I:C) on immunosuppressed colorectal cancer (CRC), particularly focusing on the stroma-rich CMS4 and the immune-enriched CMS1 subtypes, which are associated with poor prognosis and resistance to standard therapies. The authors demonstrate that poly(I:C) treatment reduces liver metastases in in vivo KPN mouse models, an effect correlated with increased infiltration of cytotoxic CD8+ T cells and activation of adaptive immune responses. In parallel in vitro analyses, the authors show that poly(I:C) induces both common and lineage-specific transcriptional programs in CRC epithelial cells and macrophages. Notably, poly(I:C) triggers cytotoxicity in some epithelial cell lines, while in macrophages, it promotes an M1-like, proinflammatory activation state—an effect validated with both bulk and single-cell transcriptomic data from human tumors. A major conceptual advance presented is the finding that poly(I:C) drives epithelial cells toward a regenerative stem-like state resembling the immune-active CMS1 subtype, while simultaneously suppressing a CMS4-associated regenerative state. The team develops and applies "poly(I:C) response" gene signatures (PRS) to both in vitro models and human tumor cohorts, revealing that CRCs can be biologically stratified along a gradient from "hot" (CMS1/dMMR-enriched, immune active), through "warm" (CMS4/pMMR, intermediate immune activity), to "cold" (CMS2/3, immune excluded). Importantly, elevated PRS scores in two independent human CRC cohorts associate with improved relapse-free survival, supporting the relevance of these signatures as potential biomarkers for clinical response.
The study is characterized by comprehensive multi-modal data integration, including use of single-cell RNA-seq to dissect macrophage phenotypes and organoid models to validate stem cell state transitions. Mechanistic insights, notably in defining lineage-specific and shared transcriptional responses, lend substantial weight to the proposition that viral mimicry could be leveraged therapeutically to reprogram the immunological landscape of high-risk CRC.

Although the manuscript shows interesting and novel findings, characterized by good clinical impact, it would benefit from revision to increase the clarity and scientific robustness of the results.

Here are the concerns of this reviewer:

- Figure 1C should include the staining (CD8) in the panel to make it clear the used antibody.
- How do the authors explain the heterogeneous results obtained by exposing cells to poly(I:C) in terms of cell viability and activation of caspases in different cancer cell lines?
- Did the authors investigate the effect of poly(I:C) on healthy cells?
- Figure 3B should include the epithelial/macrophages label on top of the specific panels to discriminate the results obtained in different cell populations.
- Figure 4E should be removed from the panel since it does not add any value to the Figure.
- Figure 5B should have included a panel showing the correlation between CBC and EP PRS to demonstrate the transition between CBC and RSC with the treatment.
- It would have been interesting to analyze in Figure 5D the common genes expressed by CMS1 and CMS4 regarding the RSC signature, to determine a shared signature between the two molecular consensus subtypes.

Reviewer #2

(Remarks to the Author)
The manuscript "Viral mimicry redirects immunosuppressed colorectal tumour landscapes towards a
2 proinflammatory and CMS1-like regenerative state" by Corry et al., describes the lineage-specific anti-metastatic responses induced by poly(I:C), enabling the development of tractable biomarkers that identify an “immune-warm” patient subset who will eventually respond to poly(I:C).

The study is well conducted. But the point is very weak.

First of all, it is totally misleading the wording "viral mimicry". This would imply an immune response directed against a viral antigen, which is not the case.

Second, it is not clear what is novel compared to the very reach literature addressing the same aspect;

Third, if these data aim to propose a clinical trial based on poly (I:C) treatment, there are many published data on the toxicity linked to poly(I:C), namely fever, weight loss, anemia, hemorrhage, and organ damage (liver necrosis, pulmonary thrombosis), and mortality.

Therefore, the interesting biological effects described in the paper are very unlikely to be useful.

Reviewer #3

(Remarks to the Author)
I find this work highly significant, and I find the proposal of making use of cell plasticity to reprogram the CMS4-warm immunosuppressive microenvironment towards a more immunogenic, CMS1-like state innovative. Overall, the manuscript presents clear and coherent arguments. I believe that this work will be impactful in helping shape future directions in the field. Comments are in the uploaded document.

Version 1:

Reviewer comments:

Reviewer #1

(Remarks to the Author)
The authors have successfully addressed the majority of the reviewers’ concerns.
The revisions have significantly improved the clarity and scientific robustness of the work.

Reviewer #2

(Remarks to the Author)

Reviewer #3

(Remarks to the Author)
Dear Editor,
Thank you for the opportunity to review the revised version of the manuscript. I have carefully examined the authors’ responses to the previous comments as well as the changes implemented in the revised manuscript.

Overall, I find that the authors have addressed the concerns raised in the initial round of review in a thorough and satisfactory manner. The revisions have improved the clarity and robustness of the study, and the manuscript is now significantly strengthened.

I only have a minor comment regarding the figures: following reviewer 1's comment, the authors added the epithelial/macrophages label on top of the specific panels in Figure 3B. However, the figure title appears to overlap slightly with the image. I suggest adjusting the layout to improve readability.
I would also encourage the authors to consider and address reviewers’ comments in future submissions.

Based on the revisions and the authors’ detailed responses, I have no further major concerns. I believe the manuscript is now suitable for publication.

Open Access This Peer Review File is licensed under a Creative Commons Attribution 4.0 International License, which permits use, sharing, adaptation, distribution and reproduction in any medium or format, as long as you give appropriate credit to the original author(s) and the source, provide a link to the Creative Commons license, and indicate if changes were made.
In cases where reviewers are anonymous, credit should be given to 'Anonymous Referee' and the source.
The images or other third party material in this Peer Review File are included in the article's Creative Commons license, unless indicated otherwise in a credit line to the material. If material is not included in the article's Creative Commons license and your intended use is not permitted by statutory regulation or exceeds the permitted use, you will need to obtain permission directly from the copyright holder.
To view a copy of this license, visit https://creativecommons.org/licenses/by/4.0/

Viral mimicry redirects immunosuppressed colorectal tumour landscapes towards a proinflammatory and CMS1-like regenerative state

Dear Editor,

We would like to thank you and the Reviewers for their time and insightful comments on our manuscript. We have read the decision letter carefully and have looked to add supporting evidence to address some of the concerns and additional questions the reviewers have highlighted. Please find below our responses to the reviewers in purple.

We appreciate the thoughtfully considered and detailed points from the Reviewers, and for the time and effort they spent providing their comments. They highlighted the way our study carried out a '*comprehensive multi-modal data integration'* to show a novel approach that will '*shape future directions in the field'*.

Reviewer #1 (Remarks to the Author):

This manuscript, submitted to Communications Biology, investigates the effects of the viral mimetic poly(I:C) on immunosuppressed colorectal cancer (CRC), particularly focusing on the stroma-rich CMS4 and the immune-enriched CMS1 subtypes, which are associated with poor prognosis and resistance to standard therapies. The authors demonstrate that poly(I:C) treatment reduces liver metastases in in vivo KPN mouse models, an effect correlated with increased infiltration of cytotoxic CD8+ T cells and activation of adaptive immune responses. In parallel in vitro analyses, the authors show that poly(I:C) induces both common and lineage-specific transcriptional programs in CRC epithelial cells and macrophages. Notably, poly(I:C) triggers cytotoxicity in some epithelial cell lines, while in macrophages, it promotes an M1-like, proinflammatory activation state—an effect validated with both bulk and single-cell transcriptomic data from human tumors.

A major conceptual advance presented is the finding that poly(I:C) drives epithelial cells toward a regenerative stem-like state resembling the immune-active CMS1 subtype, while simultaneously suppressing a CMS4-associated regenerative state.

We thank the Reviewer for recognising the novelty and conceptual importance of these findings, particular in the context of new understanding around regenerative cell states and molecular subtypes in colorectal cancer.

The team develops and applies “poly(I:C) response” gene signatures (PRS) to both in vitro models and human tumor cohorts, revealing that CRCs can be biologically stratified along a gradient from “hot” (CMS1/dMMR-enriched, immune active), through “warm” (CMS4/pMMR, intermediate immune activity), to “cold” (CMS2/3, immune excluded). Importantly, elevated PRS scores in two independent human CRC cohorts associate with improved relapse-free survival, supporting the relevance of these signatures as potential biomarkers for clinical response. The study is characterized by comprehensive multi-modal data integration, including use of single-cell RNA-seq to dissect macrophage phenotypes and organoid models to validate stem cell state transitions. Mechanistic insights, notably in defining lineage-specific and shared transcriptional responses, lend substantial weight to the proposition that viral mimicry could be leveraged therapeutically to reprogram the immunological landscape of high-risk CRC.

Again, we appreciate these comments as the goal of this study was directly related to the points described by the Reviewer.

Although the manuscript shows interesting and novel findings, characterized by good clinical impact, it would benefit from revision to increase the clarity and scientific robustness of the results. Here are the concerns of this reviewer:

- Figure 1C should include the staining (CD8) in the panel to make it clear the used antibody.

A title above Figure 1C has now been added to outline the immunohistochemistry is of CD8+ cells in liver metastasis.

- How do the authors explain the heterogeneous results obtained by exposing cells to

poly(I:C) in terms of cell viability and activation of caspases in different cancer cell lines?

The response to polyIC across any cell type relies on multiple factors and their downstream signalling cascades. The cell lines used in this analysis all have varying background mutations and phenotypes, all of which have previously been characterised and used as models of CRC, including some studies that have focussed on viability responses to poly(I:C). While the results we obtained for these cell lines in our study are heterogeneous, the use of transfection reagents, like lipofectamine, alongside poly(I:C) has been shown to increase cellular uptake and decrease cell viability PMID: 28717003.

Therefore, to address this point further in response to the Reviewers comment, we have now carried out an additional series of viability analyses on our cell lines treated with lipofectamine and poly(I:C) (below) and found this result consistent with literature.

We have added the results below for this rebuttal letter only, as we feel it would dilute the focus of our findings in the cell lines and our previous in vivo study that were all treated with poly(I:C) without transfection method.

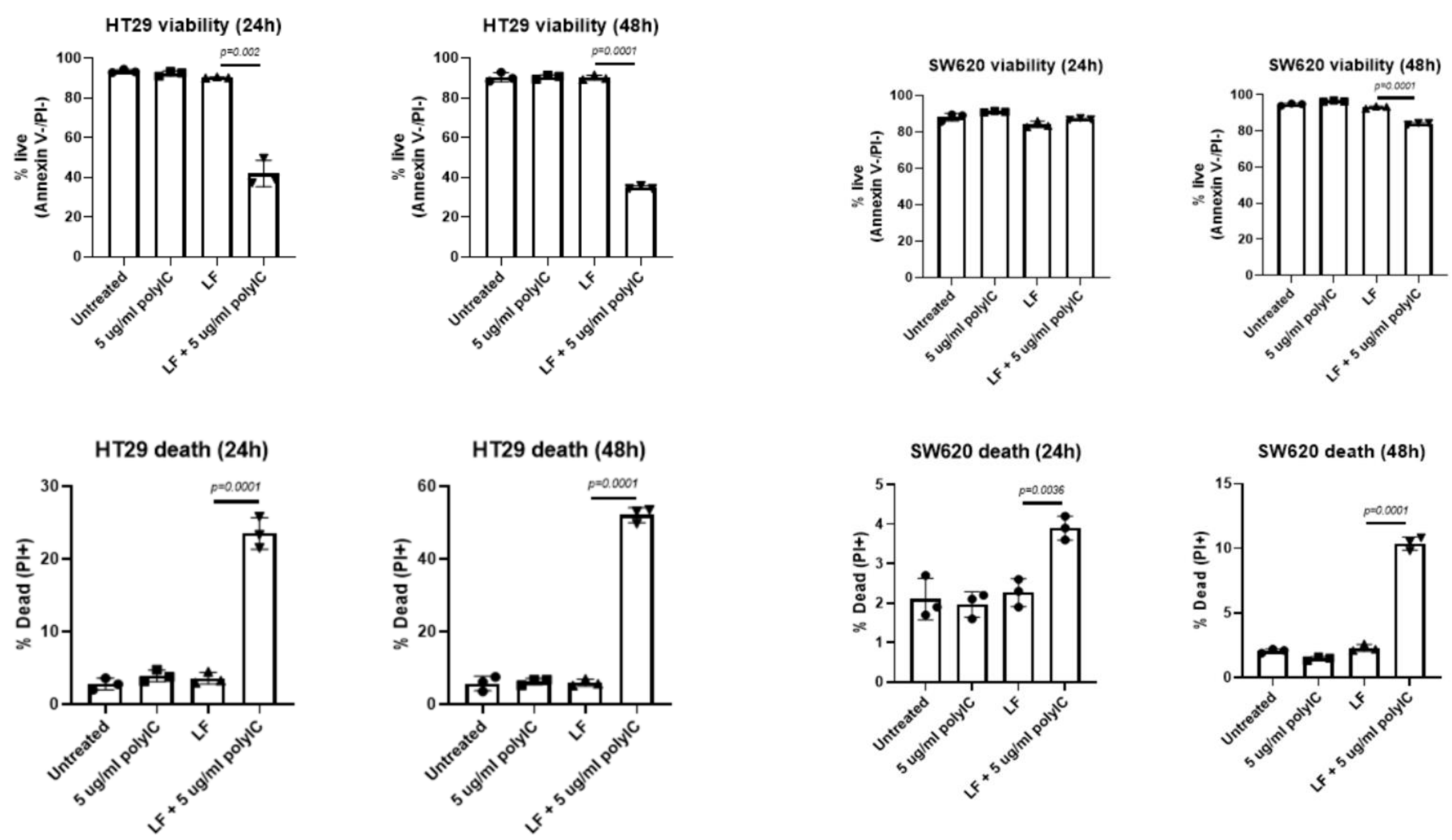

- Did the authors investigate the effect of poly(I:C) on healthy cells?

We did not look at poly(I:C) in healthy cells. This is similar to a point raised by Reviewer 2, around potential toxicities, which is further expanded on later in this rebuttal.

- Figure 3B should include the epithelial/macrophages label on top of the specific panels to discriminate the results obtained in different cell populations.

We agree with the Reviewer, this would help provide clarity. This label has been added to figure 3B.

- Figure 4E should be removed from the panel since it does not add any value to the Figure.

We agree, we have now removed the figure.

- **Figure 5B should have included a panel showing the correlation between CBC and EP PRS to demonstrate the transition between CBC and RSC with the treatment.**

As we know from the many papers published over the past 5 years in particular, there are multiple *stem phenotypes, most of which are thought to sit within the spectrum defined by the 'CBC' and 'RSC' signatures (PMID: 35931031). While these CBC/RSC states represent opposing ends of this stem spectrum, an increase in one state within a tumour sample does not always directly drive a decrease in the other state; a point described as stem cell admixtures (generating the ISCindex score, CBC < RSC) in the originally article referenced above.*

In our study, we find a strong correlation between RSC and Epithelial PRS (shown in the figure), where CMS1/4 tumours have the highest RSC and epithelial PRS, while CMS2/3 having the lowest for both signatures. As the goal of our study was to therapeutically "push" a CMS4/stroma-rich tumour to become more like a CMS1/immune-rich phenotype, we kept our focus on the RSC as it was equally enriched in both subtypes.

In new data (presented below) to address the Reviewers point, we can reveal that unlike its relationship with RSC, the epithelial poly(I:C) treatment response signature is not associated with the CBC phenotype. It is, as outlined in the initial submission, significantly enriching for a CMS1 RSC phenotype at the expense of a CMS4 RSC phenotype; a point that strongly supports the therapeutic value of poly(I:C) in this setting.

This is now included in the revised manuscript as Supplementary Figure 5B.

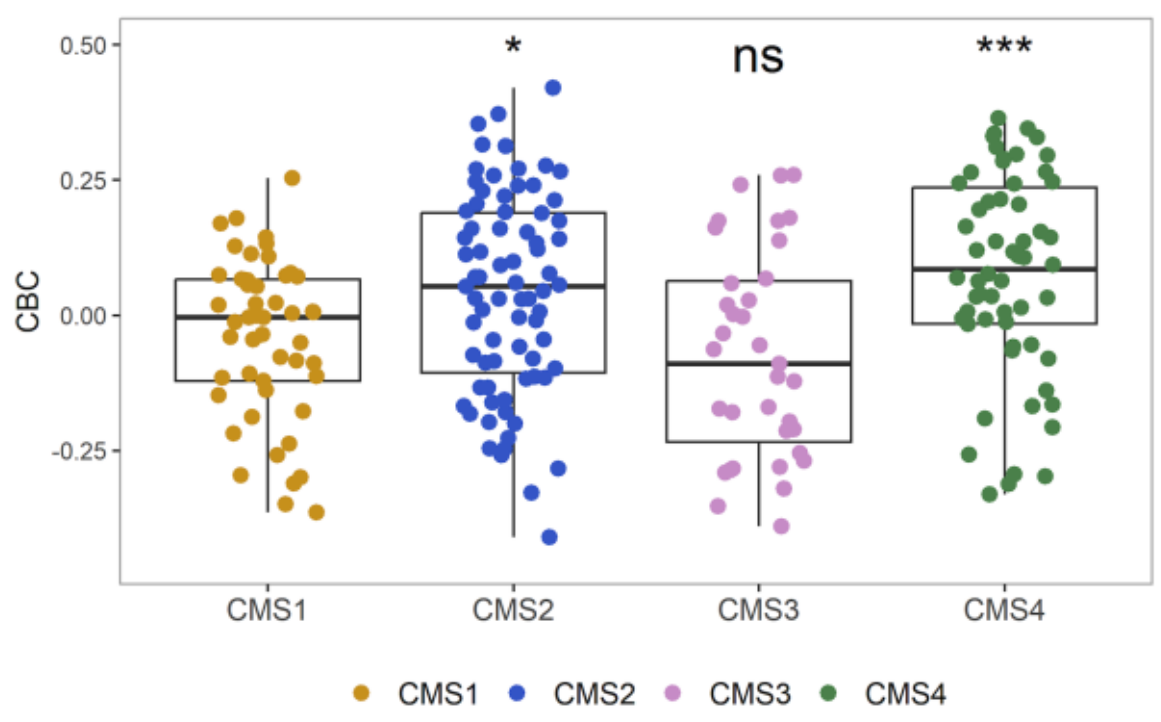

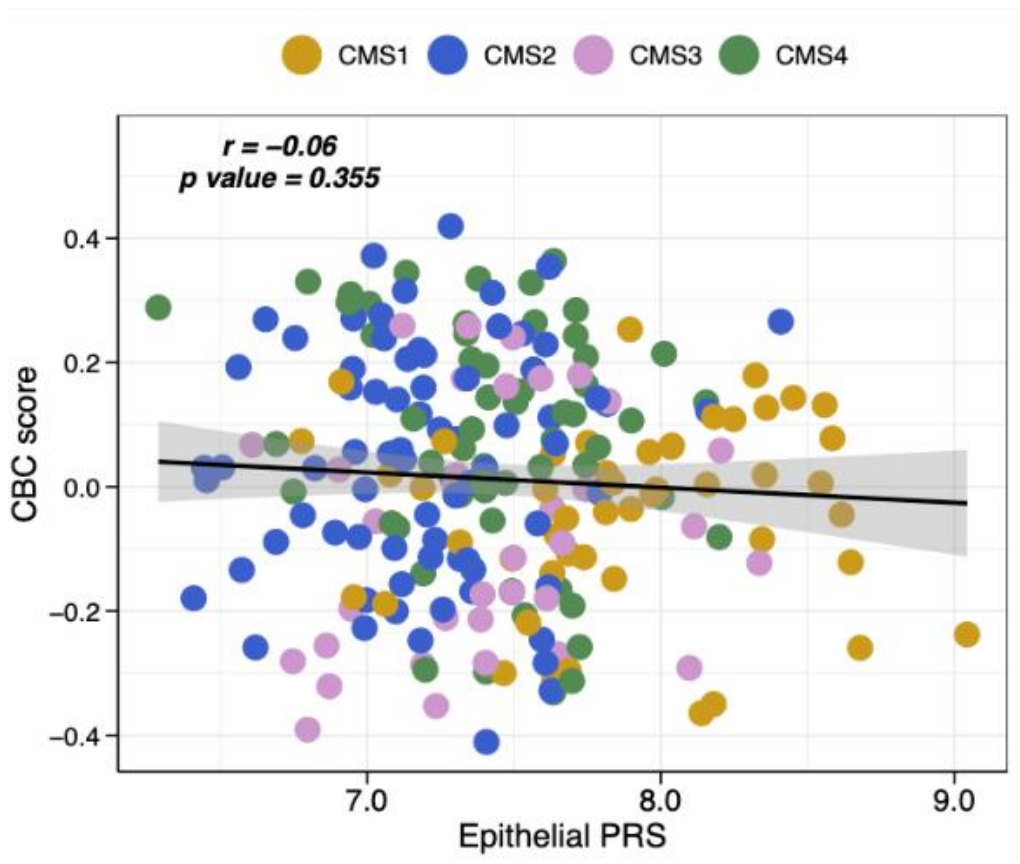

- It would have been interesting to analyze in Figure 5D the common genes expressed by CMS1 and CMS4 regarding the RSC signature, to determine a shared signature between the two molecular consensus subtypes.

We thank the Reviewer for this useful suggestion. To address this point, we identified genes that are commonly expressed by CMS1 and CMS4 using a DGEA in the same manner we had already presented in the initial submission, however this time CMS1 and CMS4 tumours were grouped against the remaining samples. Following statistical filtering (adjusted p value < 0.05) and overlapping the results with the RSC gene list in the same way as before, this new approach identified n=82 genes as being significantly enriched in CMS1 and CMS4 relative to remaining samples (shown in heatmap below). However, considering that some of these genes may be bias to either CMS1 or CMS4, we removed genes that were part of either the CMS1 RSC or CMS4 RSC gene lists, resulting in a final n=36 CMS1+CMS4 RSC gene list.

The biology of these shared genes is highlighted as being involved in 'negatively regulating apoptosis', as well as broad immune pathways such as IL-2/STAT5 signalling. This shared RSC-biology can be compared to the CMS1-RSC which is TNF-alpha, interferon enriched and to the CMS4-RSC which is EMT, MAPK enriched. This reiterates that despite both CMS's being significantly enriched for RSC phenotype relative to the remaining samples, the CMS1 and 4 RSC specific gene lists are quite distinct and can provide greater granularity to the specific underlying RSC phenotype.

To avoid confusion about these lists, we suggest having this data remain specifically for the rebuttal and not to be included in the main manuscript as we feel the broad shared RSC genes provide no further insight at this time.

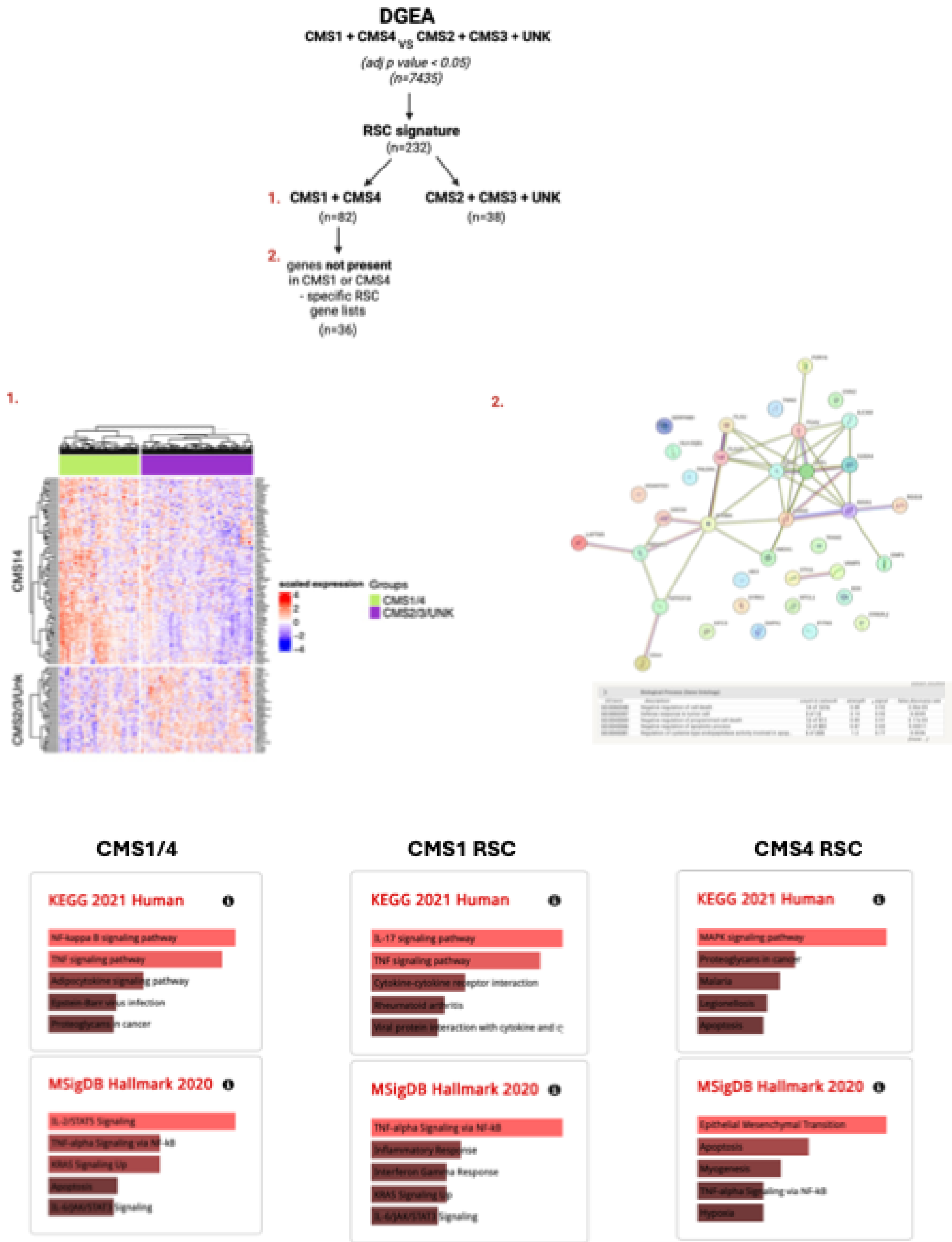
DGEA
CMS1 + CMS4 vs CMS2 + CMS3 + UNK
(adj p value < 0.05)
(n=7435)
RSC signature
(n=232)
1. CMS1 + CMS4
(n=82)
CMS2 + CMS3 + UNK
(n=38)
2. genes not present in CMS1 or CMS4 - specific RSC gene lists
(n=36)
1.
CMS14
CMS2/3/UNK
scaled expression
4
2
0
-2
-4
Groups
CMS1/4
CMS2/3/UNK
2.
CMS1/4
KEGG 2021 Human
NF-kappa B signaling pathway
TNF signaling pathway
Adipocytokine signaling pathway
Epstein-Barr virus infection
Proteoglycans in cancer
MSigDB Hallmark 2020
IL-2/STAT5 Signaling
TNF-alpha Signaling via NF-kB
KRAS Signaling Up
Apoptosis
IL-6/JAK/STAT3 Signaling
CMS1 RSC
KEGG 2021 Human
IL-17 signaling pathway
TNF signaling pathway
Cytokine-cytokine receptor interaction
Rheumatoid arthritis
Viral protein interaction with cytokine and c
MSigDB Hallmark 2020
TNF-alpha Signaling via NF-kB
Inflammatory Response
Interferon Gamma Response
KRAS Signaling Up
IL-6/JAK/STAT3 Signaling
CMS4 RSC
KEGG 2021 Human
MAPK signaling pathway
Proteoglycans in cancer
Malaria
Legionellosis
Apoptosis
MSigDB Hallmark 2020
Epithelial Mesenchymal Transition
Apoptosis
Myogenesis
TNF-alpha Signaling via NF-kB
Hypoxia

Reviewer #2 (Remarks to the Author):

The manuscript "Viral mimicry redirects immunosuppressed colorectal tumour landscapes towards a proinflammatory and CMS1-like regenerative state" by Corry et al., describes the lineage-specific anti-metastatic responses induced by poly(I:C), enabling the development of tractable biomarkers that identify an “immune-warm” patient subset who will eventually respond to poly(I:C). The study is well conducted. But the point is very weak.

First of all, it is totally misleading the wording "viral mimicry". This would imply an immune response directed against a viral antigen, which is not the case.

We thank the reviewer for this point. The emergence of canonical definitions for this response has been described previously (PMID: 26317465), which established the “viral mimicry” concept for epigenetic therapies, and many articles and reviews since then use the exact phrase to refer to activation of antiviral signalling by endogenous or synthetic dsRNA. In the literature “viral mimicry” is widely used to describe activation of innate antiviral sensing and downstream type-I interferon (IFN) signalling by double-stranded RNA (dsRNA). The terminology is still used whether that dsRNA is exogenous (e.g. the synthetic dsRNA poly(I:C)) or endogenous (e.g. dsRNA derived from reactivated transposable elements after epigenetic therapy). The term does not imply recognition of a specific viral antigen or the presence of replicating virus but rather denotes engagement of antiviral pattern-recognition receptors (e.g. TLR3, MDA5, RIG-I) and induction of an antiviral/IFN transcriptional programs; the latter of which is the focus of our study.

Below, we provide the Reviewer with additional examples of the “viral mimicry” term being used to describe these process in the cancer/innate-immunity literature, including PMCID: PMC10993856 , PMID: 40581966, PMID: 37934086, PMID: 37454216, PMID: 26317465.

In the context of our study, we have used “viral mimicry” in this same way, as the cellular state changes and responses induced by poly(I:C) that resemble the transcriptional/innate signalling changes seen in viral infection. This is deliberately distinct from saying there is a viral antigen or virus replication.

Second, it is not clear what is novel compared to the very reach literature addressing the same aspect.

As outlined by Reviewer 1 and Reviewer 3, there are a number of important novel findings in our study, most notably:

- *The conceptual advance where poly(I:C) drives epithelial cells toward a regenerative stem-like state resembling the immune-active CMS1 subtype, while simultaneously suppressing a CMS4-associated regenerative state.*
- *Improved mechanistic understanding in defining lineage-specific and shared transcriptional responses to poly(I:C).*
- *Translational value that supports how poly(I:C) could be leveraged therapeutically to reprogram the immunological landscape of high-risk CRC.*

Third, if these data aim to propose a clinical trial based on poly (I:C) treatment, there are many published data on the toxicity linked to poly(I:C), namely fever, weight loss, anemia, hemorrhage, and organ damage (liver necrosis, pulmonary thrombosis), and mortality.

Therefore, the interesting biological effects described in the paper are very unlikely to be useful.

We again thank the Reviewer for raising safety concerns about poly(I:C)-type agents. We agree that historical, high-dose systemic administration of unmodified poly(I:C) in the 1980s produced severe cytokine-related toxicities in some settings; this historical record is well documented. However, modern clinical development has mostly used poly-ICLC (Hiltonol), which is a chemically stabilised, clinical-grade TLR3/MDA5 agonist. Many contemporary clinical and translational studies using this poly(I:C) compound have reported predominantly transient, low-grade systemic effects (fever, fatigue, injection-site reactions, flu-like symptoms) and a favourable safety profile when used as vaccine adjuvant, intratumoural immune stimulant, or at the doses and schedules employed in current trials.

This is exemplified in a very recent study using poly-ICLC PMID: 41173002, Prostate cancer in situ autovaccination with the intratumoral viral mimic poly-ICLC: Modulating the cold tumor microenvironment, which had safety and tolerability of sequential intratumoural and intramuscular poly-ICLC as a primary endpoint. This study reported that this treatment was not only safe, but that it also increased immune infiltration and transcriptional changes that align with our study.

Serious organ-toxicities (e.g., liver necrosis, pulmonary thrombosis, fatal cytokine storm) that were previously associated with high-dose poly(I:C) regimens, have not been reported with modern poly-ICLC regimens in controlled clinical trials. For example, systematic and clinical reports (PMID: 34172082, PMID: 31024557) and multiple recent phase I/II studies and vaccine trials using poly-ICLC (PMID: 33479025, PMID: 33751208) document only mostly grade 1–2 AEs attributable to the agent, with very few treatment-related grade ≥3 events. These modern poly-ICLC formulations, administered intratumourally or at adjuvant doses and schedules used in recent trials, have a substantially different clinical tolerability profile from the high-dose systemic poly(I:C) regimens of early eras.

It is therefore scientifically defensible to propose translational work or a carefully designed early-phase clinical trial using poly-ICLC.

Reviewer #3 (Remarks to the Author):

I find this work highly significant, and I find the proposal of making use of cell plasticity to reprogram the CMS4-warm immunosuppressive microenvironment towards a more immunogenic, CMS1-like state innovative. Overall, the manuscript presents clear and coherent arguments. I believe that this work will be impactful in helping shape future directions in the field. Comments are in the uploaded document.

The article reports very interesting results on a novel CMS4-switch-therapy approach. CMS4 colorectal cancer subgroup includes a huge proportion of patients (around 23% according to Guinney J. et al., Nat Med. 2015;21(11):1350-1356) for whom, at the moment, a very limited number of therapies are available. Given that, I found this work of extreme importance since it finely proposes to exploit cancer cell plasticity to switch the immunosuppressive microenvironment of CMS4-warm patients towards an immunogenic, favourable CMS1-like phenotype. Here are some minor revisions:

Line 62: classifier should be classified

We have rectified this in the text.

Line 112: In this first results paragraph, I feel like the authors should spend some additional words to explain a bit more about Figure 1A. Indeed, without reading their previously published paper (Corry SM. et al., Gut 2022), grasping the meaning of the first few sentences is challenging.

We thank the reviewer for this comment and we have now edited the manuscript to add more context to the results section related to Figure 1, in order to help a reader that may not have read our previous publication.

Also, has the poly(I:C) effect been evaluated only in the metastatic tissue? Is there any effect on the primary tumour?

The point made by the reviewer around the effect of poly(I:C) on the primary tumour is of great interest and is currently part of our plan going forward, however this is not something that we have at this time.

Line 134: Given the high heterogeneity of CRC cell lines, can you point out if there is a reason why you chose HCT116 cells?

We thank the Reviewer for highlighting the important issue of CRC cell line heterogeneity. We fully agree that colorectal cancer cell lines span a wide spectrum of molecular features, and that careful justification of model choice is essential.

HCT116 cells were selected for several reasons. First, they are among the most extensively characterised CRC cell lines at the genomic, transcriptomic, and functional levels, making them a well-annotated and reproducible model for mechanistic studies. Second, HCT116 cells demonstrate robust innate immune sensing machinery, including functional TLR3/MDA5–type I interferon signalling, and importantly respond to poly(I:C) without the requirement for transfection reagents. This feature was particularly relevant for our study, as our in vivo

experiments utilised poly(I:C) without a delivery vehicle; therefore, using a cell line that exhibits direct responsiveness allowed better alignment between our in vitro and in vivo systems.

Importantly, HCT116 was not used in isolation. We included additional CRC cell lines with differing molecular characteristics (e.g., MSS backgrounds) to demonstrate the heterogeneity of viability responses, and we complemented these experiments with transcriptomic analyses in macrophages and in vivo KPN models that more faithfully recapitulate a stroma-rich CMS4 phenotype. Therefore, HCT116 served as a tractable and biologically responsive epithelial model for mechanistic dissection, rather than as the sole representation of CRC biology.

We agree that extending these analyses to additional CMS-defined or stroma-rich organoid and in vivo models will further strengthen the translational scope of this work, and this forms part of our planned follow-up studies.

Line 140: Your work starts from in vivo data, where you demonstrate the effect of poly(I:C) in reducing the metastatic tumor burden. Moving to the in vitro, did you try to evaluate the poly(I:C) effect on cells' migration and invasion capability? In HT29, SW620, and Colo-205 cells, the viability is unaffected, but did you check if the poly(I:C) somehow affects the EMT? Additionally, do you think there is a reason why the two affected cell lines (HCT116 and GP5D) are MSI, while the unaffected are all MSS?

These are fantastic points raised by the reviewer, and ultimately, we did not further evaluate the phenotype change with regards to EMT in the treated cell lines, however as EMT signatures are included in the Hallmarks database used for general characterisation, if this was a key driving biology we would have expected it to emerge at a top hit at that stage (it was well down the list in terms of enrichment scores; Supplementary Figure 3). Our in vitro analysis, solely served as a tool to gain further granularity into the core signalling pathways initially identified in our initial study such as interferon and NFkB signalling, and identify if they were differentially activated in an epithelial or immune cell.

Potentially there are mechanistic reasons underlying the reason the two sensitive cells we outline are MSI and the resistant seem to be MSS, however this would need further analysis and is outside the scope of the study.

Line 181: Should explain what RELA is

This has now been clarified in the appropriate place.

Line 285: I have a question on the effect of poly(I:C) in the epithelial cell line. The cell line you used (HCT116),in some papers, is classified as CMS4-like (Berg, KCG et al., Molecular cancer, 2017, 16,1 116; Mazzeschi M et al., J Exp Clin Cancer Res, 2022 vol. 41,1 113). However, unlike most CMS4, it presents with an MSI phenotype, which is instead typical of CMS1 patients. Do you think the similarity between the biology induced by poly(I:C) and that of CMS1 tumors could be due to the microsatellite instability present in HCT116 cells? Are there plans to investigate the effects of poly(I:C) also in cell lines that exhibit a more typical CMS4-like phenotype?

We thanks the reviewer for these points, however given some issues discussed above with cell line heterogeneity, we believe that moving this mechanistic investigation into more advanced immunocompetent mouse models represent a more comparable system to test the

acute tissue specific responses alongside more widespread systematic responses following poly(I:C) in tumour bearing mice. The plans are to use similar non-Apc stroma-rich mouse genotypes that we currently use, like KPN that recapitulate the CMS4 phenotype, alongside more conventional Apc-drive aggressive tumour models (AKP) and those with microsatellite instability mutations (Mlh1), to cover both MSS/MSI and CMS1/CMS4 landscapes, rather than having to choose between panels of cell lines.

Linee 299: Do you think that, in the future, the PRS score could be used as a biomarker to select the patients that could benefit from the poly(I:C) treatment?

Initially no, as we believe that as all stroma-rich tumour will be immune-suppressed to some extent, there may be a benefit to poly(I:C) for all. That said, we would expect the greatest benefit to be gained in patient populations that have a lower PRS, something that would require retrospective analysis of responders v non-responders in such a clinical trial.

Overall, I found the manuscript to be very well-written, with clear and logical sentences. I greatly appreciated the manuscript, even if it has the limitation that the data obtained are only related to a single epithelial cell line. As a potential follow-up, I think that it would be interesting to explore a possible combination between poly(I:C) treatment and ICIs, which might be effective after the CMS4-to-CMS1 switch.

We thank the reviewers again for these positive comments, and recognition of the value of this work despite study limitations. We are at an advanced stage of trial protocol development for such a trial, which would incorporate these combinations in an attempt to translate these findings into the clinical setting.
