# Supplementary material for: Viral mimicry redirects immunosuppressed colorectal tumour landscapes towards a proinflammatory and CMS1-like regenerative state
Source: Commun Biol. 2026 May 26;9:1119. doi: 10.1038/s42003-026-10295-9 (PMC13486658; doi:10.1038/s42003-026-10295-9)
Supplement: Supplementary file 2 [file 42003_2026_10295_MOESM2_ESM.pdf]

Supplementary Figure 1

A

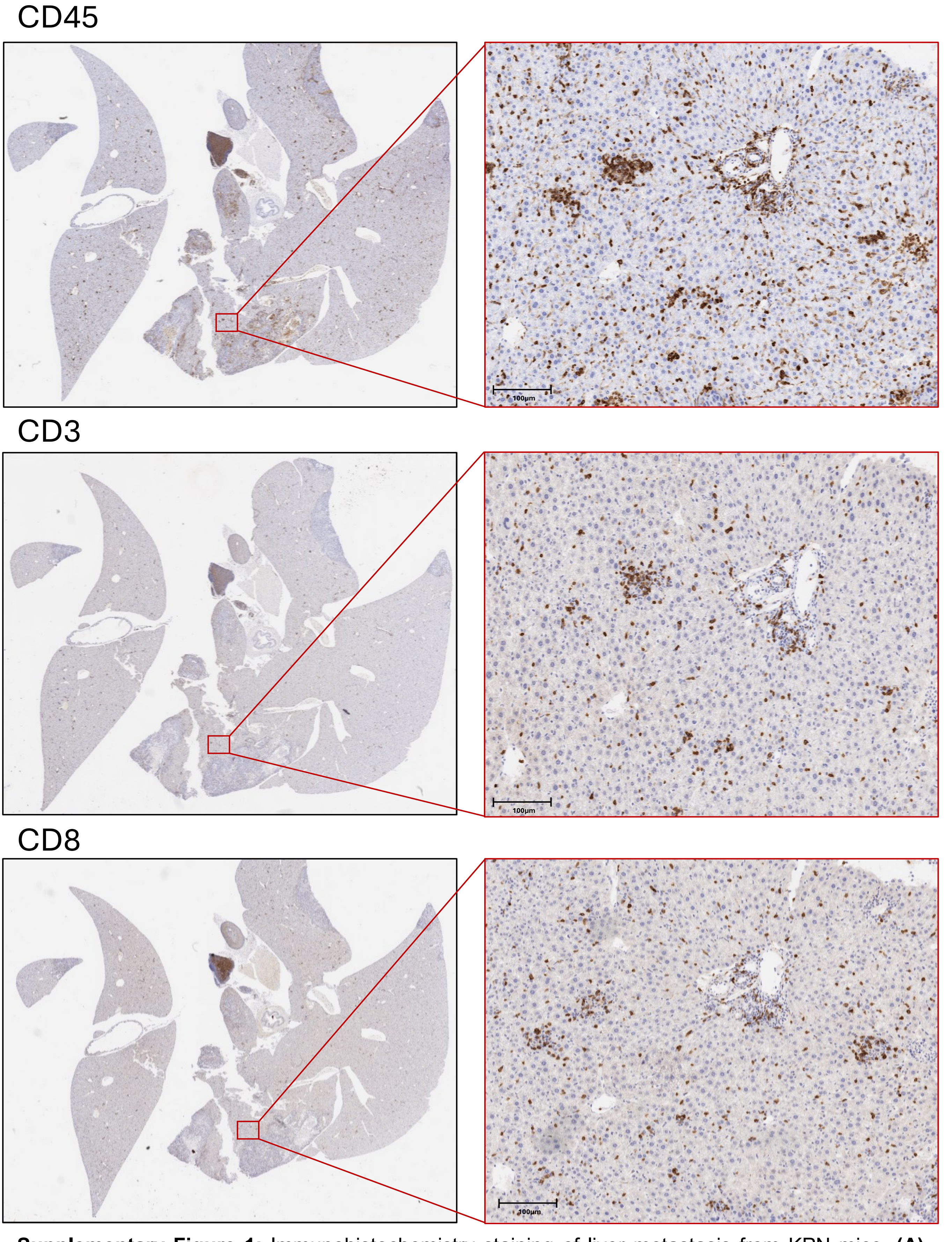

Supplementary Figure 1: Immunohistochemistry staining of liver metastasis from KPN mice. **(A)** Immunohistochemistry staining of liver sections from KPN mice treated with poly(I:C) of CD45, CD3 and CD8.

Supplementary Figure 2

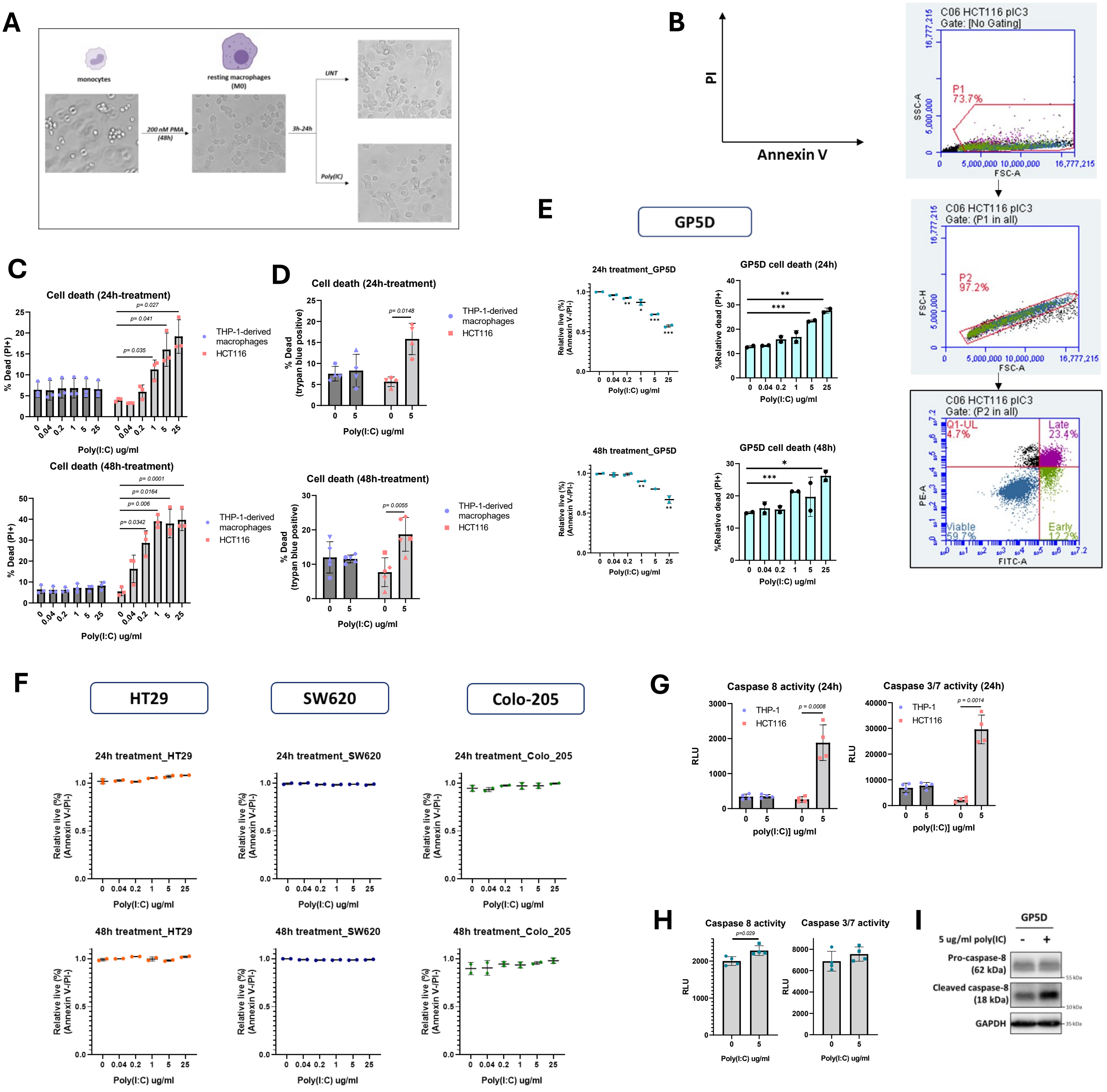

Supplementary Figure 2: Cell lineage specific response to poly(I:C). **(A)** Differentiation process of monocytes into resting macrophages. **(B)** Gating strategy for cell death quantified using flow cytometry. **(C-D)** Cell death quantification across different poly(I:C) doses at 24 and 48hrs post poly(I:C) treatment (Student t-test, carried out on Prism). **(E)** Cell death quantification at 24hrs (top) and 48hrs (bottom) following the administration of poly(I:C) to GP5D cells. **(F)** Cell death quantification at 24hrs (top) and 48hrs (bottom) of HT29, SW620 and Colo-205 epithelial cell lines. **(G)** Caspase 8 and Caspase3/7 activity at 24hrs in treated and untreated macrophage (THP-1) and epithelial (HCT116). **(H)** Caspase 8 and Caspase 3/7 activity in poly(I:C) treated and untreated GP5D cells. **(I)** Pro- and cleaved- caspase 8 in poly(I:C) treated and untreated GP5D cells. (Student t-test carried out on Prism: ns: $p > 0.05$, *: $p \le 0.05$, **: $p \le 0.01$, ***: $p \le 0.001$, ****: $p \le 0.0001$). Error bars = 1 standard deviation.

Supplementary Figure 3

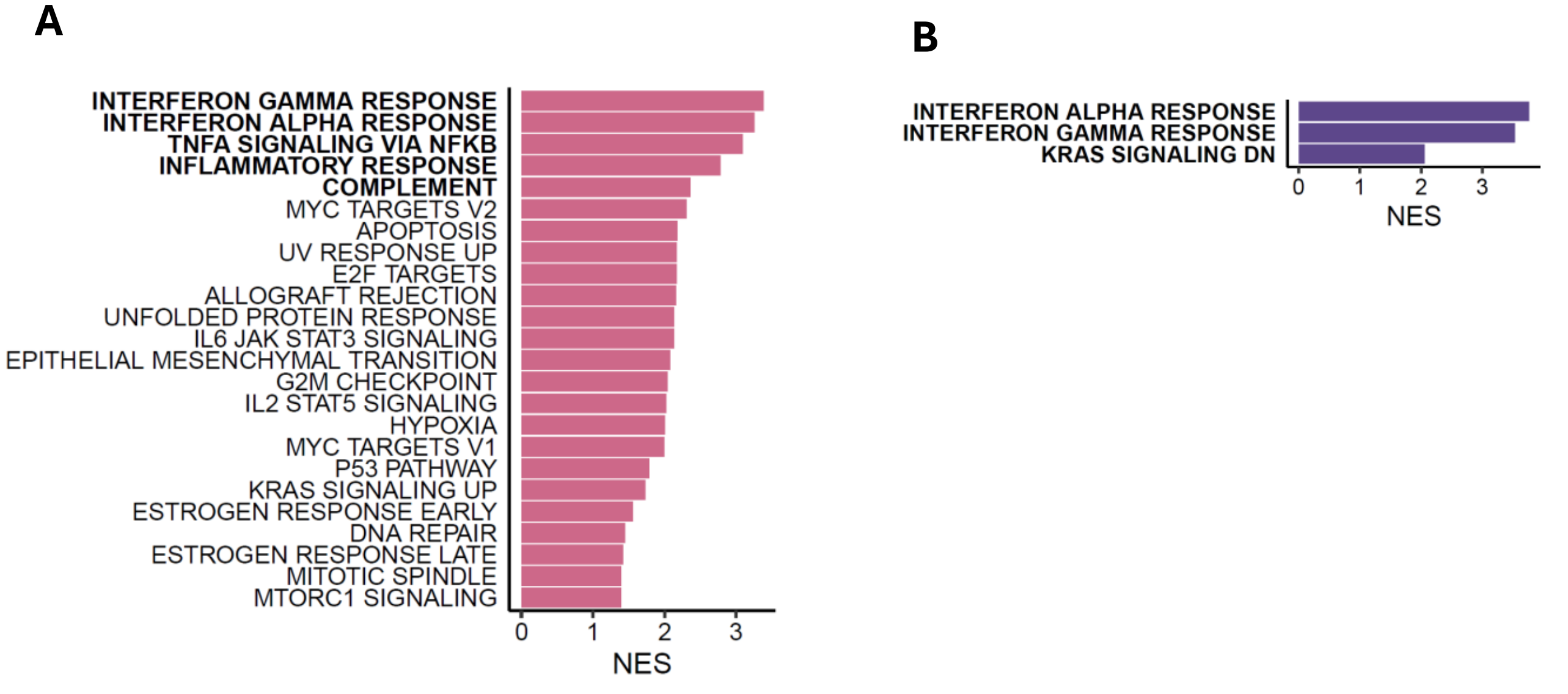

Supplementary Figure 3: Transcriptional analysis of *in vitro* model. **(A)** Gene set enrichment analysis (GSEA) of the significant Hallmark gene sets (adjusted p value < 0.05) in epithelial and **(B)** macrophage cells treated with poly(I:C) relative to their untreated controls, organised by normalized enrichment score with the top n=5 (n=3 for macrophage due to low number of significant gene sets) in bold font.

Supplementary Figure 4

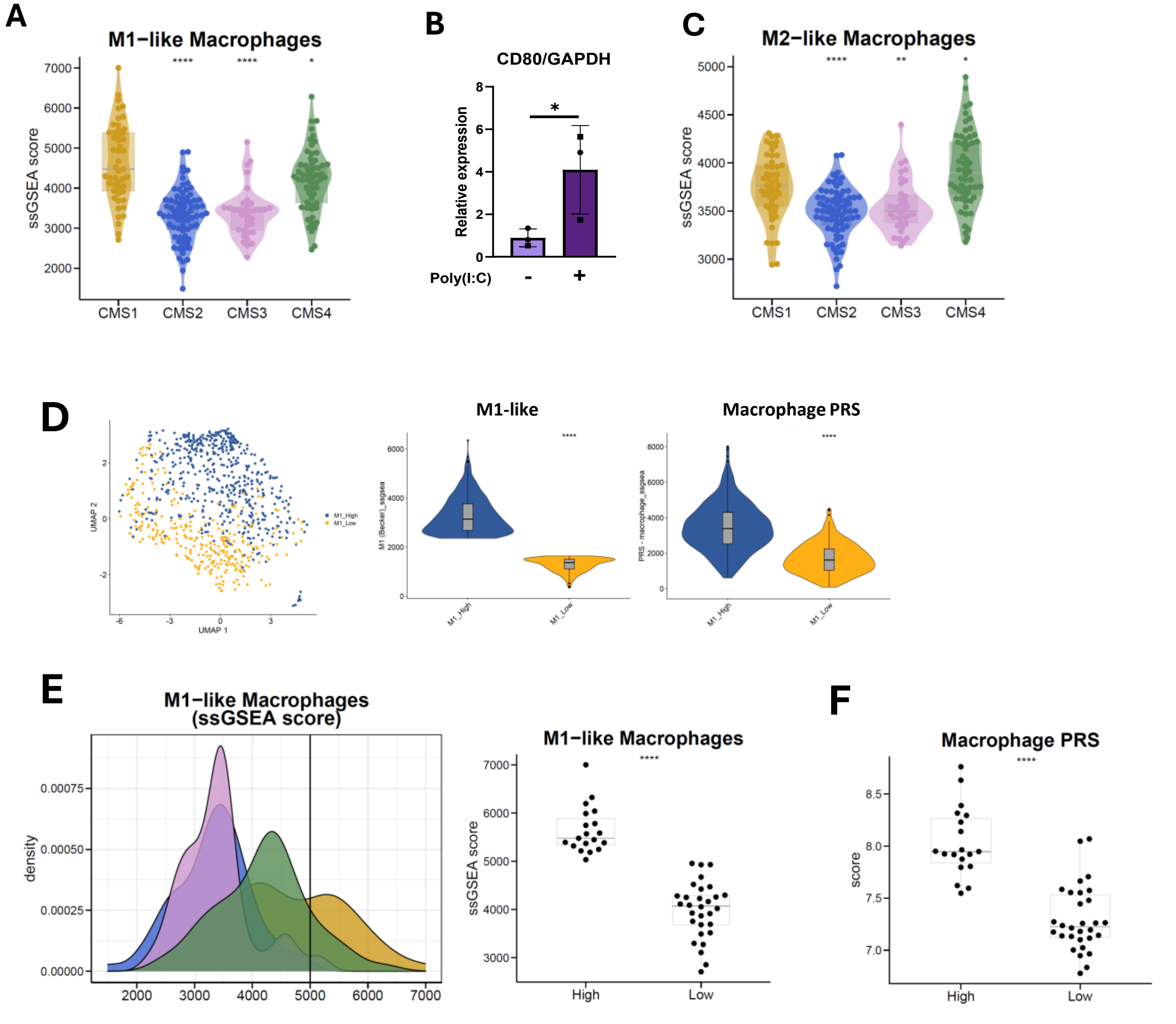

Supplementary Figure 4: Poly(I:C) treatment induces an M1-like phenotype that is encapsulated in the macrophage PRS. **(A)** M1-like transcriptional signature single sample gene set enrichment analysis (ssGSEA) scores across the CMS's (GSE39582) (n=258) (Wilcoxon rank sum test, CMS1 as reference group). **(B)** CD80 quantitative polymerase chain reaction (qPCR) between the poly(I:C) treated and untreated macrophages (Student t-test carried out on Prism: p <= 0.05). Error bars = 1 standard deviation. **(C)** M2-like transcriptional signature single sample gene set enrichment analysis (ssGSEA) scores across the CMS's (Wilcoxon rank sum test, CMS1 as reference group). **(D)** The myeloid subpopulation from the single cell RNA-sequencing cohort (GSE132465) (n=23) that were split into tertiles based on M1 and M2 transcriptional scores, with the top (M1 high) and bottom (M1 low) tertile visualised (left) and compared based on their M1-like transcriptional score and macrophage PRS score (right) (Wilcoxon rank sum test). **(E)** M1-like transcriptional signature ssGSEA scores across the CMS's visualised as a ridge plot (left), with a vertical line creating a stratification point into M1 high and M1 low of CMS1 samples (right). **(F)** The macrophage PRS in the M1 high subset of CMS1 samples compared to the M1 low subset of CMS1 samples. (Wilcoxon rank sum carried out by ggpubr: ns: p > 0.05, *: p <= 0.05, **: p <= 0.01, ***: p <= 0.001, ****: p <= 0.0001).

Supplementary Figure 5

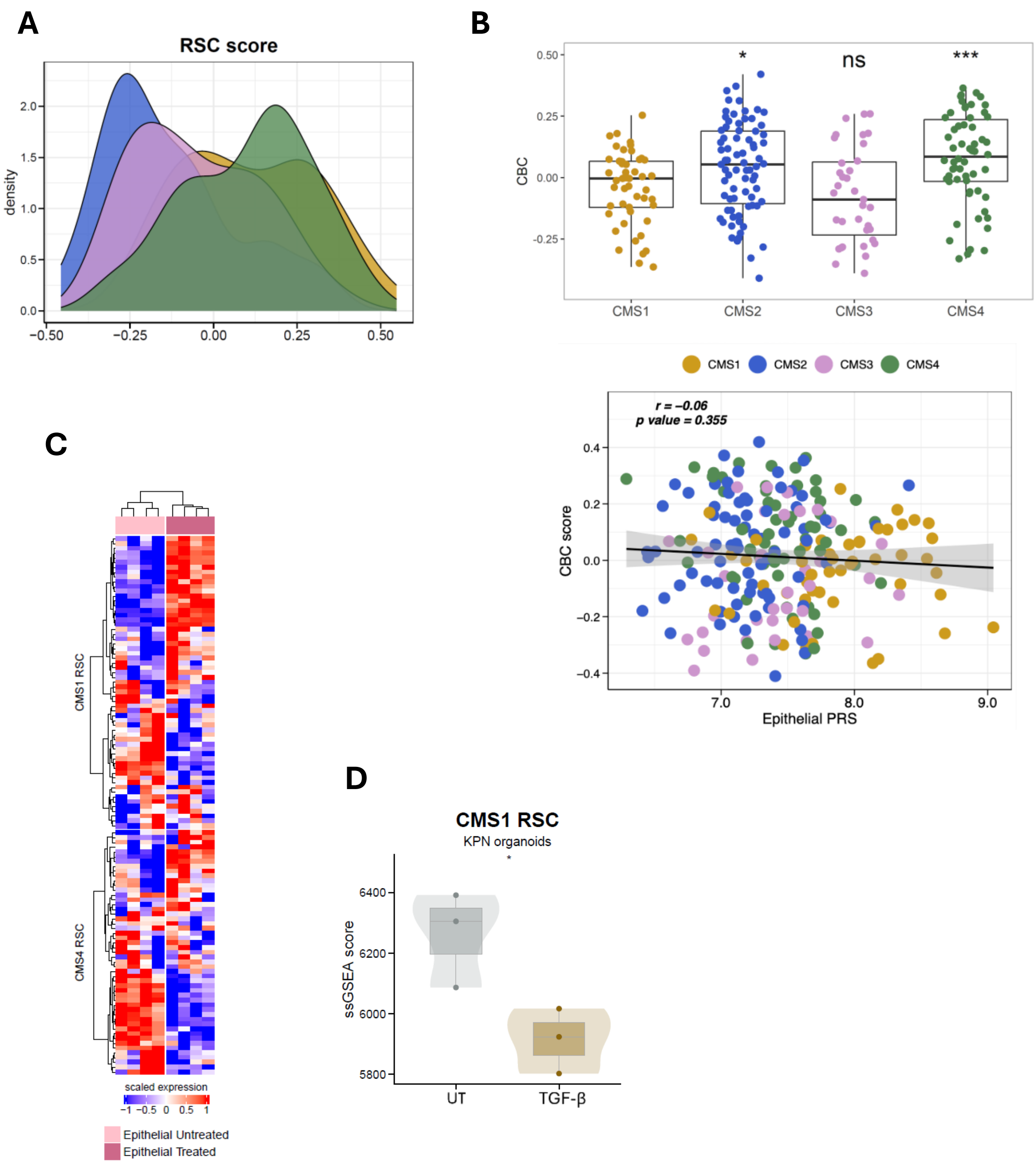

Supplementary Figure 5: Poly(I:C) induces a CMS1-like regenerative stem phenotype. **(A)** Regenerative stem cell (RSC) signature score across the CMS's visualised in a ridge plot. **(B)** Columnar base cell (CBC) signature across the CMS's (Wilcoxon rank sum test, CMS1 as reference group) (top) and Pearsons correlation of CBC and epithelial PRS (bottom). Both (A) and (B) using cohort (GSE39582) (n=258). (Wilcoxon rank sum carried out by ggpubr: ns: p > 0.05, *: p <= 0.05, **: p <= 0.01, ***: p <= 0.001, ****: p <= 0.0001). **(C)** The genes from the CMS1 RSC and CMS4 RSC visualised across the poly(I:C)-treated and untreated epithelial cells. **(D)** ssGSEA scores of CMS1 RSC in KPN Organoids treated with TGF-β (E-MTAB-11784) (t-test, p value; * <= 0.05).

Supplementary Figure 6

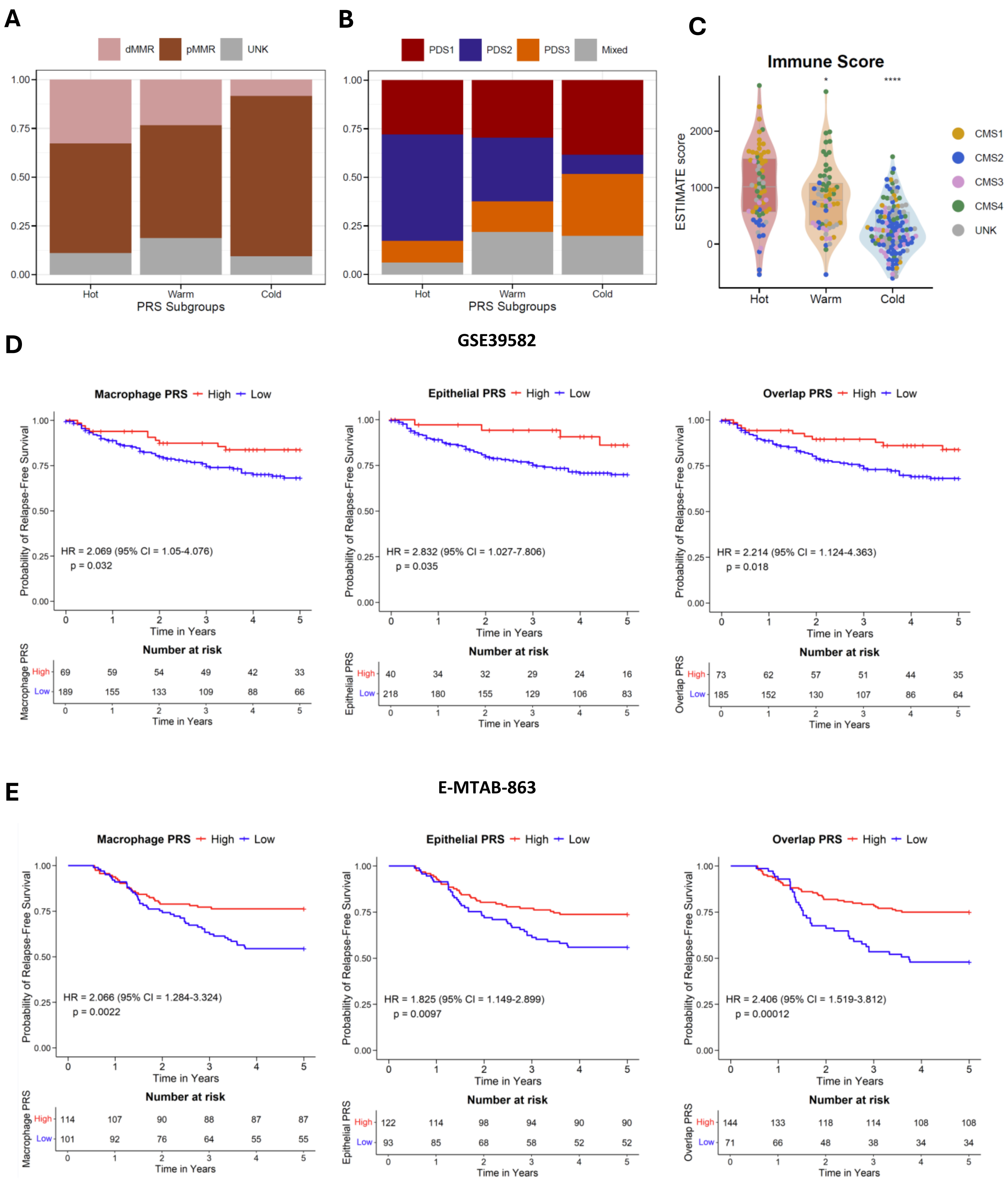

Supplementary Figure 6: Poly(I:C) biology aligns with an immune active CMS1-like phenotype. **(A)** Proportion of mismatch repair deficient (d) and proficient (p) samples across the PRS subgroups (GSE39582) (n=258). **(B)** Proportions of samples classified as the pathway derived subtypes (PDS) within the PRS subgroups. **(C)** ESTIMATE immune score across the PRS subgroups (Wilcoxon rank sum test with Hot subgroup as reference). **(D)** Kaplan-Meier survival estimates of macrophage, epithelial and overlap PRS in the GSE39582 cohort (n=258) and **(E)** an independent stage II colon cancer cohort (n=215) (E-MTAB-863) (all significant, p value < 0.05). (Wilcoxon rank sum carried out by ggpubr: ns: p > 0.05, *: p <= 0.05, **: p <= 0.01, ***: p <= 0.001, ****: p <= 0.0001).

Supplementary Figure 7

Full blots from Figure 2F

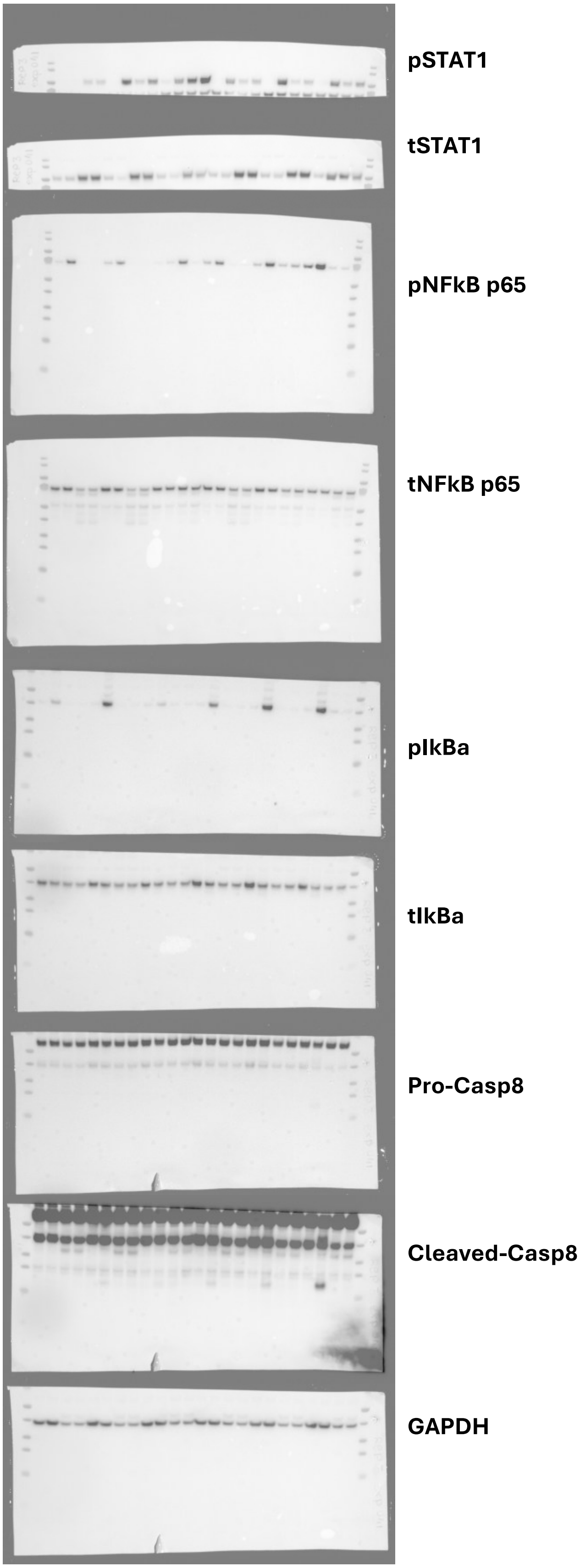

Full blots from Figure 2G

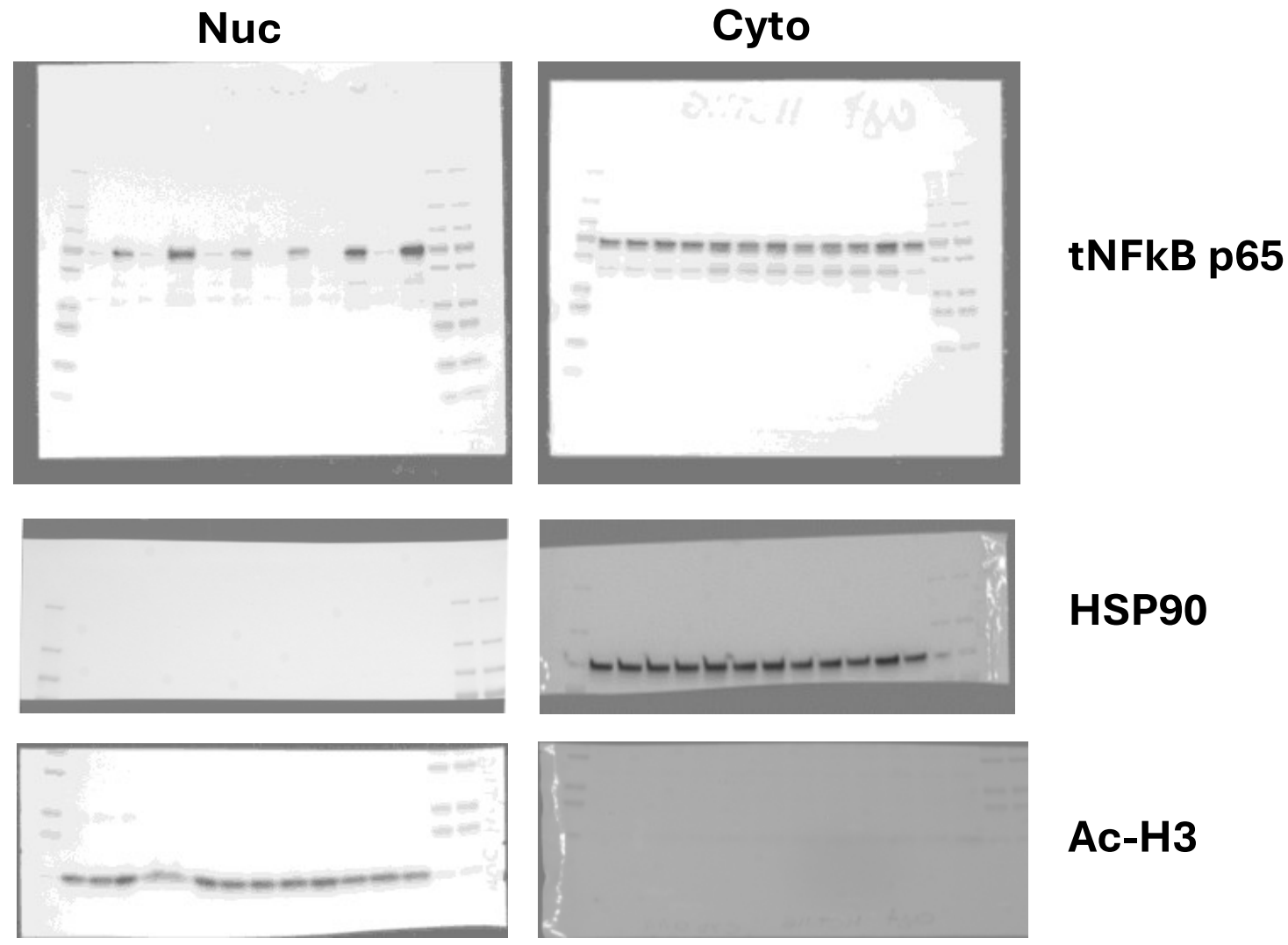

Full blots from Figure 2H

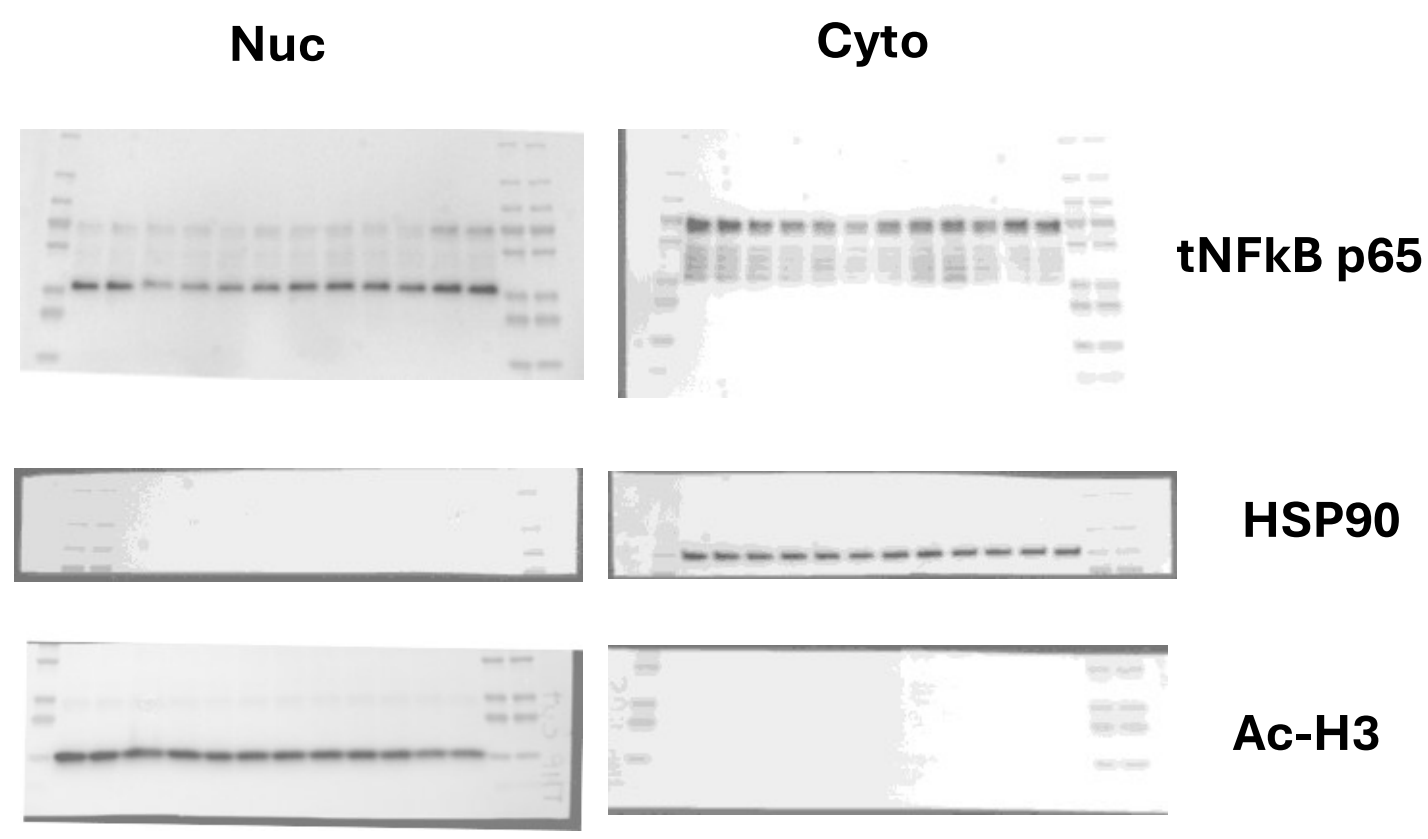

Full blots from Figure 4D

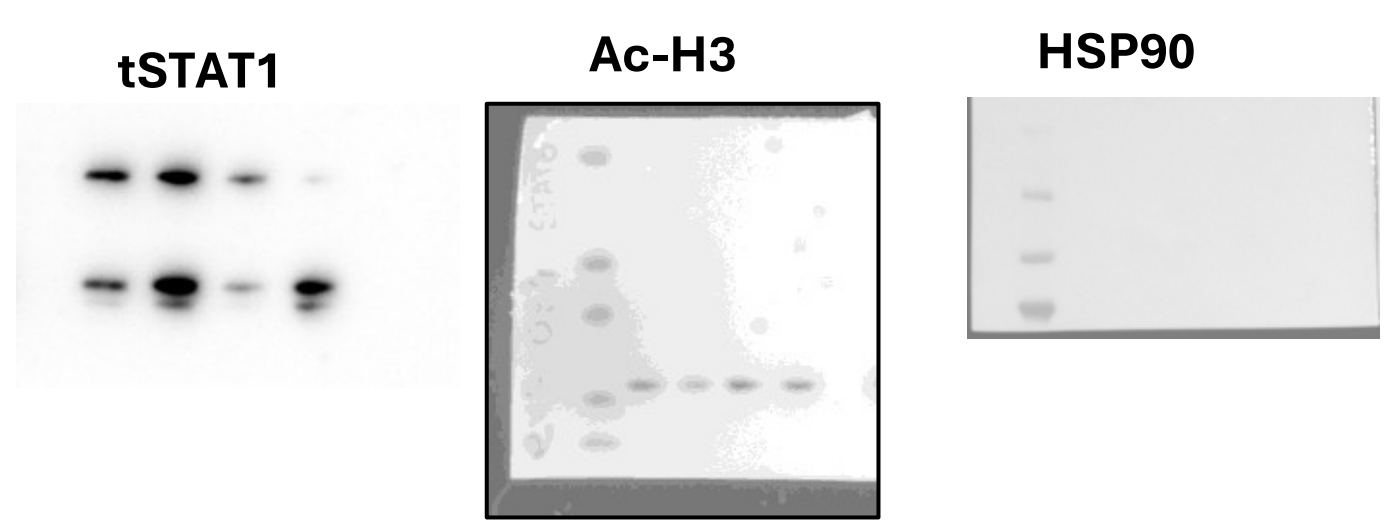

Full blots from Supp Figure 2I

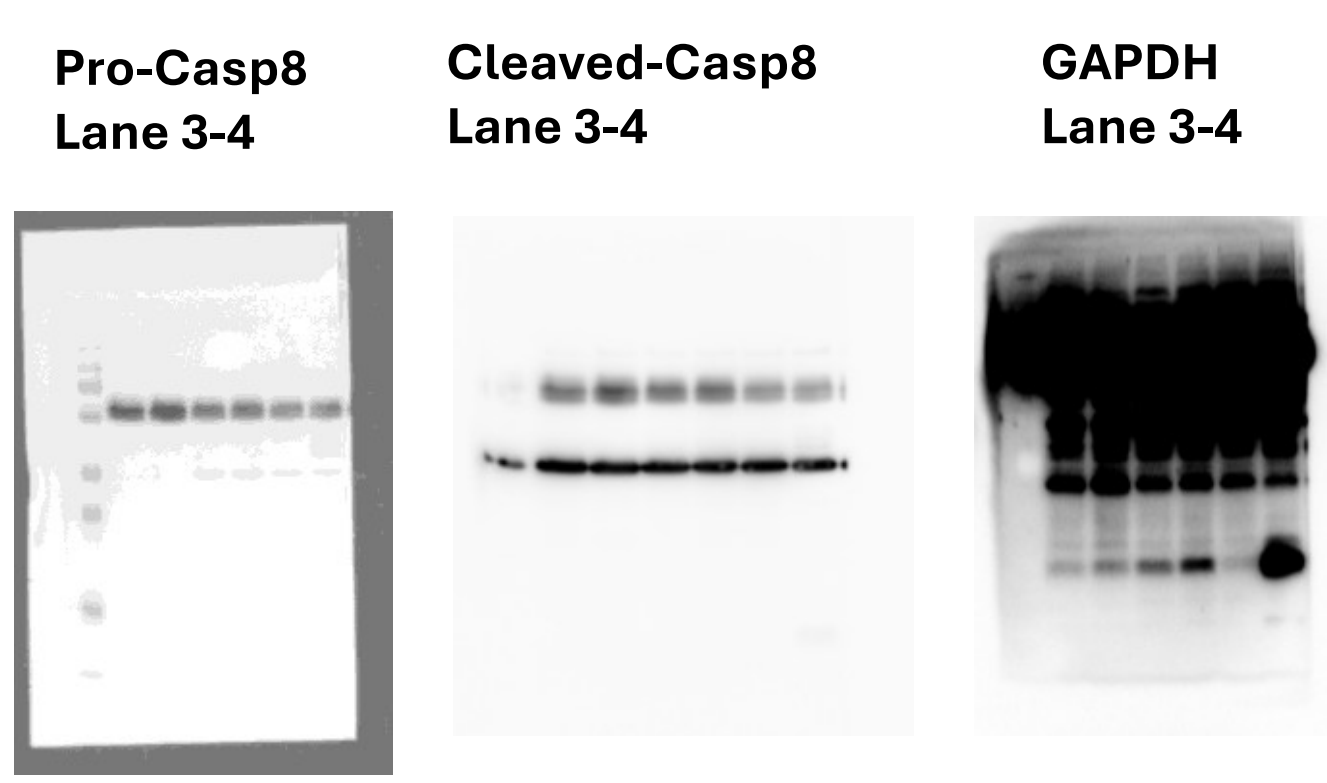

Supplementary Figure 7: Uncropped blots from manuscript figures.
