# Supplementary material for: Viral mimicry redirects immunosuppressed colorectal tumour landscapes towards a proinflammatory and CMS1-like regenerative state
Source: Commun Biol. 2026 May 26;9:1119. doi: 10.1038/s42003-026-10295-9 (PMC13486658; doi:10.1038/s42003-026-10295-9)
Supplement: Supplementary file 3 [file 42003_2026_10295_MOESM3_ESM.pdf]

Description of Additional Supplementary files

File name: Supplementary Data 1

Description: The source data behind the graphs in the paper.
