# Supplementary material for: Viral mimicry redirects immunosuppressed colorectal tumour landscapes towards a proinflammatory and CMS1-like regenerative state
Source: Commun Biol. 2026 May 26;9:1119. doi: 10.1038/s42003-026-10295-9 (PMC13486658; doi:10.1038/s42003-026-10295-9)
Supplement: Supplementary file 4 [file 42003_2026_10295_MOESM5_ESM.pdf]

nature portfolio

Corresponding author(s): Philip Dunne, Shania Corry

Last updated by author(s): May 6, 2026

# Reporting Summary

Nature Portfolio wishes to improve the reproducibility of the work that we publish. This form provides structure for consistency and transparency in reporting. For further information on Nature Portfolio policies, see our Editorial Policies and the Editorial Policy Checklist.

## Statistics

For all statistical analyses, confirm that the following items are present in the figure legend, table legend, main text, or Methods section.

| n/a | Confirmed | |
|---|---|---|
| ☐ | ☒ | The exact sample size (*n*) for each experimental group/condition, given as a discrete number and unit of measurement |
| ☐ | ☒ | A statement on whether measurements were taken from distinct samples or whether the same sample was measured repeatedly |
| ☐ | ☒ | The statistical test(s) used AND whether they are one- or two-sided<br>*Only common tests should be described solely by name; describe more complex techniques in the Methods section.* |
| ☐ | ☒ | A description of all covariates tested |
| ☐ | ☒ | A description of any assumptions or corrections, such as tests of normality and adjustment for multiple comparisons |
| ☐ | ☒ | A full description of the statistical parameters including central tendency (e.g. means) or other basic estimates (e.g. regression coefficient) AND variation (e.g. standard deviation) or associated estimates of uncertainty (e.g. confidence intervals) |
| ☐ | ☒ | For null hypothesis testing, the test statistic (e.g. *F*, *t*, *r*) with confidence intervals, effect sizes, degrees of freedom and *P* value noted<br>*Give P values as exact values whenever suitable.* |
| ☒ | ☐ | For Bayesian analysis, information on the choice of priors and Markov chain Monte Carlo settings |
| ☒ | ☐ | For hierarchical and complex designs, identification of the appropriate level for tests and full reporting of outcomes |
| ☐ | ☒ | Estimates of effect sizes (e.g. Cohen's *d*, Pearson's *r*), indicating how they were calculated |

*Our web collection on statistics for biologists contains articles on many of the points above.*

## Software and code

Policy information about availability of computer code

| | |
|---|---|
| Data collection | There is no new code or relevant material generated in this study. |
| Data analysis | There is no new code or relevant material generated in this study. |

For manuscripts utilizing custom algorithms or software that are central to the research but not yet described in published literature, software must be made available to editors and reviewers. We strongly encourage code deposition in a community repository (e.g. GitHub). See the Nature Portfolio guidelines for submitting code & software for further information.

## Data

Policy information about availability of data

All manuscripts must include a data availability statement. This statement should provide the following information, where applicable:

- Accession codes, unique identifiers, or web links for publicly available datasets
- A description of any restrictions on data availability
- For clinical datasets or third party data, please ensure that the statement adheres to our policy

The accession numbers for the publicly available bulk and single cell gene expression dataset used in this study for both the human and mouse models are all referred to in the Methods, with corresponding GEO accession numbers. The new poly(I:C)-treated datasets from HCT116 and THP-1 lineages are available on GEO, under accession number GSE280541.

## Research involving human participants, their data, or biological material

Policy information about studies with human participants or human data. See also policy information about sex, gender (identity/presentation), and sexual orientation and race, ethnicity and racism.

| | |
|---|---|
| Reporting on sex and gender | N/A |
| Reporting on race, ethnicity, or other socially relevant groupings | N/A |
| Population characteristics | N/A |
| Recruitment | N/A |
| Ethics oversight | N/A |

Note that full information on the approval of the study protocol must also be provided in the manuscript.

# Field-specific reporting

Please select the one below that is the best fit for your research. If you are not sure, read the appropriate sections before making your selection.

☒ Life sciences ☐ Behavioural & social sciences ☐ Ecological, evolutionary & environmental sciences

For a reference copy of the document with all sections, see nature.com/documents/nr-reporting-summary-flat.pdf

# Life sciences study design

All studies must disclose on these points even when the disclosure is negative.

| | |
|---|---|
| Sample size | Samples sizes are described throughtout |
| Data exclusions | N/A |
| Replication | Replication is described throughout |
| Randomization | N/A |
| Blinding | N/A |

# Behavioural & social sciences study design

All studies must disclose on these points even when the disclosure is negative.

| | |
|---|---|
| Study description | *Briefly describe the study type including whether data are quantitative, qualitative, or mixed-methods (e.g. qualitative cross-sectional, quantitative experimental, mixed-methods case study).* |
| Research sample | *State the research sample (e.g. Harvard university undergraduates, villagers in rural India) and provide relevant demographic information (e.g. age, sex) and indicate whether the sample is representative. Provide a rationale for the study sample chosen. For studies involving existing datasets, please describe the dataset and source.* |
| Sampling strategy | *Describe the sampling procedure (e.g. random, snowball, stratified, convenience). Describe the statistical methods that were used to predetermine sample size OR if no sample-size calculation was performed, describe how sample sizes were chosen and provide a rationale for why these sample sizes are sufficient. For qualitative data, please indicate whether data saturation was considered, and what criteria were used to decide that no further sampling was needed.* |
| Data collection | *Provide details about the data collection procedure, including the instruments or devices used to record the data (e.g. pen and paper, computer, eye tracker, video or audio equipment) whether anyone was present besides the participant(s) and the researcher, and whether the researcher was blind to experimental condition and/or the study hypothesis during data collection.* |
| Timing | *Indicate the start and stop dates of data collection. If there is a gap between collection periods, state the dates for each sample cohort.* |
| Data exclusions | *If no data were excluded from the analyses, state so OR if data were excluded, provide the exact number of exclusions and the rationale behind them, indicating whether exclusion criteria were pre-established.* |

| | |
|---|---|
| Non-participation | *State how many participants dropped out/declined participation and the reason(s) given OR provide response rate OR state that no participants dropped out/declined participation.* |
| Randomization | *If participants were not allocated into experimental groups, state so OR describe how participants were allocated to groups, and if allocation was not random, describe how covariates were controlled.* |

# Ecological, evolutionary & environmental sciences study design

All studies must disclose on these points even when the disclosure is negative.

| | |
|---|---|
| Study description | *Briefly describe the study. For quantitative data include treatment factors and interactions, design structure (e.g. factorial, nested, hierarchical), nature and number of experimental units and replicates.* |
| Research sample | *Describe the research sample (e.g. a group of tagged Passer domesticus, all Stenocereus thurberi within Organ Pipe Cactus National Monument), and provide a rationale for the sample choice. When relevant, describe the organism taxa, source, sex, age range and any manipulations. State what population the sample is meant to represent when applicable. For studies involving existing datasets, describe the data and its source.* |
| Sampling strategy | *Note the sampling procedure. Describe the statistical methods that were used to predetermine sample size OR if no sample-size calculation was performed, describe how sample sizes were chosen and provide a rationale for why these sample sizes are sufficient.* |
| Data collection | *Describe the data collection procedure, including who recorded the data and how.* |
| Timing and spatial scale | *Indicate the start and stop dates of data collection, noting the frequency and periodicity of sampling and providing a rationale for these choices. If there is a gap between collection periods, state the dates for each sample cohort. Specify the spatial scale from which the data are taken* |
| Data exclusions | *If no data were excluded from the analyses, state so OR if data were excluded, describe the exclusions and the rationale behind them, indicating whether exclusion criteria were pre-established.* |
| Reproducibility | *Describe the measures taken to verify the reproducibility of experimental findings. For each experiment, note whether any attempts to repeat the experiment failed OR state that all attempts to repeat the experiment were successful.* |
| Randomization | *Describe how samples/organisms/participants were allocated into groups. If allocation was not random, describe how covariates were controlled. If this is not relevant to your study, explain why.* |
| Blinding | *Describe the extent of blinding used during data acquisition and analysis. If blinding was not possible, describe why OR explain why blinding was not relevant to your study.* |

Did the study involve field work? ☐ Yes ☐ No

## Field work, collection and transport

| | |
|---|---|
| Field conditions | *Describe the study conditions for field work, providing relevant parameters (e.g. temperature, rainfall).* |
| Location | *State the location of the sampling or experiment, providing relevant parameters (e.g. latitude and longitude, elevation, water depth).* |
| Access & import/export | *Describe the efforts you have made to access habitats and to collect and import/export your samples in a responsible manner and in compliance with local, national and international laws, noting any permits that were obtained (give the name of the issuing authority, the date of issue, and any identifying information).* |
| Disturbance | *Describe any disturbance caused by the study and how it was minimized.* |

# Reporting for specific materials, systems and methods

We require information from authors about some types of materials, experimental systems and methods used in many studies. Here, indicate whether each material, system or method listed is relevant to your study. If you are not sure if a list item applies to your research, read the appropriate section before selecting a response.

## Materials & experimental systems

| n/a | Involved in the study | |
|---|---|---|
| ☐ | ☒ | Antibodies |
| ☐ | ☒ | Eukaryotic cell lines |
| ☒ | ☐ | Palaeontology and archaeology |
| ☐ | ☒ | Animals and other organisms |
| ☐ | ☒ | Clinical data |
| ☒ | ☐ | Dual use research of concern |
| ☒ | ☐ | Plants |

## Methods

| n/a | Involved in the study | |
|---|---|---|
| ☒ | ☐ | ChIP-seq |
| ☐ | ☒ | Flow cytometry |
| ☒ | ☐ | MRI-based neuroimaging |

# Antibodies

Antibodies used

The following Western blot antibodies were used: Phospho NF-κB p65/RelA (S536) (RRID: AB_331284), Phospho IκBα (S32) (RRID: AB_561111), IκBα (RRID: AB_390781), STAT1 (RRID: AB_2198300), STAT2 (RRID: AB_2271323) (all Cell Signalling Technology); GAPDH (Abcam, RRID: AB_2107448), NF-κB p65 (Total RelA) (Santa Cruz Biotechnology , RRID: AB_628017), ; Acetyl Histone H3 (EMD Millipore); Caspase-8 (both Enzo Life Sciences) (RRID: AB_2050949), pSTAT1 (Y701) (Invitrogen, ThermoFisher) (RRID: AB_2533113).

In vivo immunohistochemistry. All Immunohistochemistry (IHC) staining was performed on 4μm formalin fixed paraffin embedded sections (FFPE) which had previously been ovened at 60ºC for 2 hours. The following antibodies were stained on a Leica Bond Rx autostainer, CD3 (ab16669, Abcam), CD8a (14-0808-82, eBioscience) and pan-CD45 (ab10558, Abcam). All FFPE sections underwent on-board dewaxing (AR9222, Leica) and epitope retrieval using ER2 retrieval solution (AR9640, Leica) for 20 minutes at 100°C. Sections were rinsed with Leica wash buffer (AR9590, Leica) before peroxidase block was performed using an Intense R kit (DS9263, Leica) for 5 minutes. Sections were rinsed with wash buffer before sections for CD8a had blocking solution applied from the Rat ImmPRESS kit (MP7444-15, Vector Labs) for 20 minutes. Sections were rinsed with wash buffer before primary antibody application at an optimal dilution (CD3, 1/100; CD8a, 1/500; pan-CD45, 1/2500). The sections were rinsed with wash buffer and then sections being stained for CD3 and pan-CD45 had rabbit envision (K4003, Agilent) applied and sections for CD8a staining had rat ImmPRESS secondary antibody applied for 30 minutes. The sections were rinsed with wash buffer, visualised using DAB and counterstained with haematoxylin in the Intense R kit. The IHC staining sections were rinsed in tap water, dehydrated through a series of graded alcohols and placed in xylene. The stained sections were coverslipped in xylene using DPX mountant (SEA-1300-00A, CellPath).

Validation

Controls and treated included in each experiment.

# Eukaryotic cell lines

Policy information about cell lines and Sex and Gender in Research

Cell line source(s)

Cells were originally sourced from ATCC and supplied for this study by co-author Longley. THP-1 (RRID: CVCL_0006), HCT116 (RRID: CVCL_0291), Colo-205 (RRID: CVCL_0218), GP5D (RRID: CVCL_1235), SW620 (RRID: CVCL_0547) and HT29 (RRID: CVCL_0320) were used. HCT116 cell lines were cultured in Dulbecco's modified Eagle's medium (DMEM) supplemented with 10% FBS, 50 U/mL penicillin, 0.1 mg/mL streptomycin, 2 mM l-glutamine, and 1 mM sodium pyruvate (all Gibco). THP-1 cells were obtained from American Type Culture Collection (ATCC, Manassas, VA, USA) and cultured in RPMI medium (Invitrogen, Paisley, UK) with 10% heat-inactivated FBS, 50 U/mL penicillin, 0.1 mg/mL streptomycin and 0.05 mM β- mercaptoethanol (Sigma). All cells were maintained at 37 °C in a 5% CO2 humidified atmosphere and regularly screened for the presence of mycoplasma using the MycoAlert Mycoplasma Detection Kit (Lonza). Cell lines were cultured for no more than 20 passages following thawing. THP-1 cells were differentiated to M0 macrophages by 48h treatment with 200nM Phorbol 12-myristate 13-acetate (Sigma-Aldrich) in complete RPMI media. After removing the media, THP-1-derived macrophages were incubated with indicated poly(I:C) concentrations in serum-free RPMI media. Poly(I:C) treatments of HCT116 cells were also performed in DMEM serum-free media.

For tissue flow cytometry, CD8 this was fluorochrome BUV805 from BD Biosciences, catalogue number 612898, CD3 PE/Dazzle594 Biolegend 100246, CD4 BUV395 BD Biosciences 563790 and CD45 AF700 Biolegend 103128 respectively.

Authentication

Routine STR typing

Mycoplasma contamination

MycoAlert Mycoplasma Detection Kit

Commonly misidentified lines
(See ICLAC register)

N/A

# Palaeontology and Archaeology

Specimen provenance

*Provide provenance information for specimens and describe permits that were obtained for the work (including the name of the issuing authority, the date of issue, and any identifying information). Permits should encompass collection and, where applicable, export.*

| | |
|---|---|
| Specimen deposition | *Indicate where the specimens have been deposited to permit free access by other researchers.* |
| Dating methods | *If new dates are provided, describe how they were obtained (e.g. collection, storage, sample pretreatment and measurement), where they were obtained (i.e. lab name), the calibration program and the protocol for quality assurance OR state that no new dates are provided.* |

☐ Tick this box to confirm that the raw and calibrated dates are available in the paper or in Supplementary Information.

| | |
|---|---|
| Ethics oversight | *Identify the organization(s) that approved or provided guidance on the study protocol, OR state that no ethical approval or guidance was required and explain why not.* |

Note that full information on the approval of the study protocol must also be provided in the manuscript.

## Animals and other research organisms

Policy information about studies involving animals; ARRIVE guidelines recommended for reporting animal research, and Sex and Gender in Research

| | |
|---|---|
| Laboratory animals | No new animal studies were performed. Data from previous studies was used however details of that animal work is included below:<br><br>Mouse experiments were performed in accordance with the UK Home Office regulations, project licence 70/9112, which had been approved by the Animal Welfare and Ethical Review Board of the University of Glasgow. We have complied with all relevant ethical regulations for animal use. All animals were monitored daily with weights taken three times a week. The animals in this study were taken at a time-point prior to clinical signs. All animals were euthanized by exposure to carbon dioxide gas in a rising concentration with the death confirmed by the secondary method of dislocation of the neck.<br>Intrasplenic injections of the cell lines included the following anaesthesia and analgesics. Carprofen in drinking water 24 pre surgery and 48 hours post surgery (0.1mg/ml). Carprofen via SC at time of surgery 4mg/kg. Buprenorphine via SC at time of surgery 0.05mg/kg. Anaesthesia used was Sevoflurane gas. Local anaesthesia used was Bupivacaine (max dose 8mg/kg) applied via splash block of surgical field prior to closing. |
| Wild animals | NA |
| Reporting on sex | Intrasplenic injection was performed in male adult C57Bl/6J mice. The organoid recipient and donor were sex and strain matched. |
| Field-collected samples | NA |
| Ethics oversight | Mouse experiments were performed in accordance with the UK Home Office regulations, project licence 70/9112, which had been approved by the Animal Welfare and Ethical Review Board of the University of Glasgow. |

Note that full information on the approval of the study protocol must also be provided in the manuscript.

## Clinical data

Policy information about clinical studies
All manuscripts should comply with the ICMJE guidelines for publication of clinical research and a completed CONSORT checklist must be included with all submissions.

| | |
|---|---|
| Clinical trial registration | N/A |
| Study protocol | *Note where the full trial protocol can be accessed OR if not available, explain why.* |
| Data collection | *Describe the settings and locales of data collection, noting the time periods of recruitment and data collection.* |
| Outcomes | *Describe how you pre-defined primary and secondary outcome measures and how you assessed these measures.* |

## Dual use research of concern

Policy information about dual use research of concern

### Hazards

Could the accidental, deliberate or reckless misuse of agents or technologies generated in the work, or the application of information presented in the manuscript, pose a threat to:

| No | Yes | |
|---|---|---|
| ☒ | ☐ | Public health |
| ☒ | ☐ | National security |
| ☒ | ☐ | Crops and/or livestock |
| ☒ | ☐ | Ecosystems |
| ☒ | ☐ | Any other significant area |

### Experiments of concern

Does the work involve any of these experiments of concern:

| No | Yes | |
|---|---|---|
| ☒ | ☐ | Demonstrate how to render a vaccine ineffective |
| ☒ | ☐ | Confer resistance to therapeutically useful antibiotics or antiviral agents |
| ☒ | ☐ | Enhance the virulence of a pathogen or render a nonpathogen virulent |
| ☒ | ☐ | Increase transmissibility of a pathogen |
| ☒ | ☐ | Alter the host range of a pathogen |
| ☒ | ☐ | Enable evasion of diagnostic/detection modalities |
| ☒ | ☐ | Enable the weaponization of a biological agent or toxin |
| ☒ | ☐ | Any other potentially harmful combination of experiments and agents |

## Plants

Seed stocks

*Report on the source of all seed stocks or other plant material used. If applicable, state the seed stock centre and catalogue number. If plant specimens were collected from the field, describe the collection location, date and sampling procedures.*

Novel plant genotypes

*Describe the methods by which all novel plant genotypes were produced. This includes those generated by transgenic approaches, gene editing, chemical/radiation-based mutagenesis and hybridization. For transgenic lines, describe the transformation method, the number of independent lines analyzed and the generation upon which experiments were performed. For gene-edited lines, describe the editor used, the endogenous sequence targeted for editing, the targeting guide RNA sequence (if applicable) and how the editor was applied.*

Authentication

*Describe any authentication procedures for each seed stock used or novel genotype generated. Describe any experiments used to assess the effect of a mutation and, where applicable, how potential secondary effects (e.g. second site T-DNA insertions, mosiacism, off-target gene editing) were examined.*

## ChIP-seq

### Data deposition

☐ Confirm that both raw and final processed data have been deposited in a public database such as GEO.

☐ Confirm that you have deposited or provided access to graph files (e.g. BED files) for the called peaks.

Data access links
*May remain private before publication.*

*For "Initial submission" or "Revised version" documents, provide reviewer access links. For your "Final submission" document, provide a link to the deposited data.*

Files in database submission

*Provide a list of all files available in the database submission.*

Genome browser session
(e.g. UCSC)

*Provide a link to an anonymized genome browser session for "Initial submission" and "Revised version" documents only, to enable peer review. Write "no longer applicable" for "Final submission" documents.*

### Methodology

Replicates

*Describe the experimental replicates, specifying number, type and replicate agreement.*

Sequencing depth

*Describe the sequencing depth for each experiment, providing the total number of reads, uniquely mapped reads, length of reads and whether they were paired- or single-end.*

Antibodies

*Describe the antibodies used for the ChIP-seq experiments; as applicable, provide supplier name, catalog number, clone name, and lot number.*

Peak calling parameters

*Specify the command line program and parameters used for read mapping and peak calling, including the ChIP, control and index files used.*

| | |
|---|---|
| Data quality | *Describe the methods used to ensure data quality in full detail, including how many peaks are at FDR 5% and above 5-fold enrichment.* |
| Software | *Describe the software used to collect and analyze the ChIP-seq data. For custom code that has been deposited into a community repository, provide accession details.* |

# Flow Cytometry

## Plots

Confirm that:

☒ The axis labels state the marker and fluorochrome used (e.g. CD4-FITC).

☒ The axis scales are clearly visible. Include numbers along axes only for bottom left plot of group (a 'group' is an analysis of identical markers).

☒ All plots are contour plots with outliers or pseudocolor plots.

☒ A numerical value for number of cells or percentage (with statistics) is provided.

## Methodology

| | |
|---|---|
| Sample preparation | Sample preparation: THP1–derived macrophages and HCT116 cells were stimulated with poly(I:C) for the indicated durations. To ensure recovery of both adherent and nonadherent (floating) cells, the entire contents of each well were collected into a single tube. First, the culture supernatant was transferred to a labelled tube to retain floating cells. The wells were then washed once with PBS, and the wash was added to the same tube. Remaining adherent cells were detached using 0.25% trypsin and transferred into the corresponding tube, ensuring that all fractions from each well were pooled. The combined cell suspensions were centrifuged at 400 × g for 5 minutes at room temperature. Resulting pellets were resuspended in 500µL of BD Binding Buffer (BD, Cat. 556454) containing FITCconjugated Annexin V (BD Pharmingen, Cat. 556419) and incubated for 15 minutes at room temperature. Immediately before acquisition, 2µL of propidium iodide (PI) was added to each sample. A minimum of 10,000 events were acquired in each experiment.<br><br>the liver metastasis samples were dissected and digested using the Mouse Tumour Dissociation Kit (Miltenyi Biotec, 130-096-730) and the GentleMACS Octo Dissociator (Miltenyi Biotec, 130-095-937), following the manufacturer's instructions for these tumours. Cells were passed through a 70µm cell strainer, washed with RPMI 1640, 10% fetal bovine serum, 2mM EDTA, and spun down at 400g, 4°C for 5 minutes. The cells were washed with PBS and spun down at 400g, 4°C for 5 minutes. After discarding the supernatant, the cells were stained with 50 µL of the Live/Dead NIR staining kit, at 1:1000 dilution in PBS, for 20min at 4°C in the dark. After that, the cells were washed with PBS 1% BSA and spun down at 400g, 4°C for 5 minutes. After discarding the supernatant, the cells were resuspended in 25µL of FcBlock (BD Pharmingen, 553141) 1:200 in PBS 1% BSA, and incubated for 10 minutes on ice in the dark. Then, 25µL of the antibody staining mix at 2x concentration were added; CD8 was fluorochrome BUV805 from BD Biosciences, catalogue number 612898, CD3 PE/Dazzle594 Biolegend 100246, CD4 BUV395 BD Biosciences 563790 and CD45 AF700 Biolegend 103128 respectively. |
| Instrument | Instrument: BD AccuriTM C6 flow cytometer |
| Software | Software: FlowJo and BD Software |
| Cell population abundance | Cell population abundance: Depending on the cell type and the poly(I:C) concentration, the proportion of viable cells (Annexin $V^-$/$PI^-$) ranged from approximately 45% to 95%, while the proportion of dead cells ($PI^+$) ranged from roughly 3% to 50%. |
| Gating strategy | Gating strategy: Cells were first gated on forward and side scatter to exclude debris and aggregates. Singlets were then selected using FSC-A/FSC-H. Within this population, viability and apoptosis were assessed by Annexin V/PI staining. PI positive cells were classified as non viable (dead). Annexin V negative/PI negative cells were classified as viable (live). Intermediate populations (Annexin V positive/PI negative were not included in the live or dead gates. |

☒ Tick this box to confirm that a figure exemplifying the gating strategy is provided in the Supplementary Information.

# Magnetic resonance imaging

## Experimental design

| | |
|---|---|
| Design type | *Indicate task or resting state; event-related or block design.* |
| Design specifications | *Specify the number of blocks, trials or experimental units per session and/or subject, and specify the length of each trial or block (if trials are blocked) and interval between trials.* |
| Behavioral performance measures | *State number and/or type of variables recorded (e.g. correct button press, response time) and what statistics were used to establish that the subjects were performing the task as expected (e.g. mean, range, and/or standard deviation across subjects).* |

## Acquisition

| | |
|---|---|
| Imaging type(s) | *Specify: functional, structural, diffusion, perfusion.* |
| Field strength | *Specify in Tesla* |
| Sequence & imaging parameters | *Specify the pulse sequence type (gradient echo, spin echo, etc.), imaging type (EPI, spiral, etc.), field of view, matrix size, slice thickness, orientation and TE/TR/flip angle.* |
| Area of acquisition | *State whether a whole brain scan was used OR define the area of acquisition, describing how the region was determined.* |

Diffusion MRI ☐ Used ☐ Not used

## Preprocessing

| | |
|---|---|
| Preprocessing software | *Provide detail on software version and revision number and on specific parameters (model/functions, brain extraction, segmentation, smoothing kernel size, etc.).* |
| Normalization | *If data were normalized/standardized, describe the approach(es): specify linear or non-linear and define image types used for transformation OR indicate that data were not normalized and explain rationale for lack of normalization.* |
| Normalization template | *Describe the template used for normalization/transformation, specifying subject space or group standardized space (e.g. original Talairach, MNI305, ICBM152) OR indicate that the data were not normalized.* |
| Noise and artifact removal | *Describe your procedure(s) for artifact and structured noise removal, specifying motion parameters, tissue signals and physiological signals (heart rate, respiration).* |
| Volume censoring | *Define your software and/or method and criteria for volume censoring, and state the extent of such censoring.* |

## Statistical modeling & inference

| | |
|---|---|
| Model type and settings | *Specify type (mass univariate, multivariate, RSA, predictive, etc.) and describe essential details of the model at the first and second levels (e.g. fixed, random or mixed effects; drift or auto-correlation).* |
| Effect(s) tested | *Define precise effect in terms of the task or stimulus conditions instead of psychological concepts and indicate whether ANOVA or factorial designs were used.* |

Specify type of analysis: ☐ Whole brain ☐ ROI-based ☐ Both

| | |
|---|---|
| Statistic type for inference (See Eklund et al. 2016) | *Specify voxel-wise or cluster-wise and report all relevant parameters for cluster-wise methods.* |
| Correction | *Describe the type of correction and how it is obtained for multiple comparisons (e.g. FWE, FDR, permutation or Monte Carlo).* |

## Models & analysis

| n/a | Involved in the study |
|---|---|
| ☐ | ☐ Functional and/or effective connectivity |
| ☐ | ☐ Graph analysis |
| ☐ | ☐ Multivariate modeling or predictive analysis |

| | |
|---|---|
| Functional and/or effective connectivity | *Report the measures of dependence used and the model details (e.g. Pearson correlation, partial correlation, mutual information).* |
| Graph analysis | *Report the dependent variable and connectivity measure, specifying weighted graph or binarized graph, subject- or group-level, and the global and/or node summaries used (e.g. clustering coefficient, efficiency, etc.).* |
| Multivariate modeling and predictive analysis | *Specify independent variables, features extraction and dimension reduction, model, training and evaluation metrics.* |
